# Mechanistic Basis for Understanding the Dual Activities of the Bifunctional Azotobacter vinelandii Mannuronan C-5-Epimerase and Alginate Lyase AlgE7

**DOI:** 10.1128/aem.01836-21

**Published:** 2022-02-08

**Authors:** Margrethe Gaardløs, Tonje Marita Bjerkan Heggeset, Anne Tøndervik, David Tezé, Birte Svensson, Helga Ertesvåg, Håvard Sletta, Finn Lillelund Aachmann

**Affiliations:** a Norwegian Biopolymer Laboratory (NOBIPOL), Department of Biotechnology and Food Science, NTNU Norwegian University of Science and Technology, Trondheim, Norway; b Department of Biotechnology and Nanomedicine, SINTEF Industry, Trondheim, Norway; c Department of Biotechnology and Biomedicine, Technical University of Denmark, Kongens Lyngby, Denmark; d Novo Nordisk Foundation Center for Biosustainability, Technical University of Denmark, Kongens Lyngby, Denmark; University of Nebraska—Lincoln

**Keywords:** alginate, alginate C-5-epimerase, alginate lyase, multifunctional enzyme, site‐directed mutagenesis, nuclear magnetic resonance (NMR), time-resolved NMR, enzyme mechanism

## Abstract

The structure and functional properties of alginates are dictated by the monomer composition and molecular weight distribution. Mannuronan C-5-epimerases determine the monomer composition by catalyzing the epimerization of β-d-mannuronic acid (M) residues into α-l-guluronic acid (G) residues. The molecular weight is affected by alginate lyases, which catalyze a β-elimination mechanism that cleaves alginate chains. The reaction mechanisms for the epimerization and lyase reactions are similar, and some enzymes can perform both reactions. These dualistic enzymes share high sequence identity with mannuronan C-5-epimerases without lyase activity. The mechanism behind their activity and the amino acid residues responsible for it are still unknown. We investigate mechanistic determinants involved in the bifunctional epimerase and lyase activity of AlgE7 from Azotobacter vinelandii. Based on sequence analyses, a range of AlgE7 variants were constructed and subjected to activity assays and product characterization by nuclear magnetic resonance (NMR) spectroscopy. Our results show that calcium promotes lyase activity, whereas NaCl reduces the lyase activity of AlgE7. By using defined polymannuronan (polyM) and polyalternating alginate (polyMG) substrates, the preferred cleavage sites of AlgE7 were found to be M|XM and G|XM, where X can be either M or G. From the study of AlgE7 mutants, R148 was identified as an important residue for the lyase activity, and the point mutant R148G resulted in an enzyme with only epimerase activity. Based on the results obtained in the present study, we suggest a unified catalytic reaction mechanism for both epimerase and lyase activities where H154 functions as the catalytic base and Y149 functions as the catalytic acid.

**IMPORTANCE** Postharvest valorization and upgrading of algal constituents are promising strategies in the development of a sustainable bioeconomy based on algal biomass. In this respect, alginate epimerases and lyases are valuable enzymes for tailoring the functional properties of alginate, a polysaccharide extracted from brown seaweed with numerous applications in food, medicine, and material industries. By providing a better understanding of the catalytic mechanism and of how the two enzyme actions can be altered by changes in reaction conditions, this study opens further applications of bacterial epimerases and lyases in the enzymatic tailoring of alginate polymers.

## INTRODUCTION

Alginate-modifying enzymes, and in particular mannuronan C-5-epimerases and lyases, can be used industrially to tailor the functional properties of alginate for food, medicine, and material purposes (1–4). Postpolymerization modifications by epimerases and lyases determine alginate applications because they affect the viscosity and gel-forming abilities (5). Alginate is a linear polysaccharide consisting of the C-5-epimers β-d-mannuronic acid (M) and α-l-guluronic acid (G), which occur in nonrandom block patterns of consecutive G residues (G blocks), consecutive M residues (M blocks), and alternating MG residues (MG blocks) (6, 7). M residues are present in a ^4^C_1_ chair conformation, whereas G residues take on a ^1^C_4_ conformation. The distinct structures of G blocks and, to a small extent, MG blocks allow the cross-linking of chains in junction zones coordinated by divalent ions like calcium, causing gel formation (8). The M/G block composition is important for the viscosity and gel properties of the alginate. Mannuronan C-5-epimerases catalyze the polymer-level epimerization of M residues into G residues (9). Alginate is harvested from brown algae, but the most-studied alginate-modifying enzymes are from alginate-producing species of Pseudomonas and *Azotobacter* (10, 11). Two main types of bacterial epimerases are characterized: the periplasmic, calcium-independent AlgG and the extracellular, calcium-dependent AlgEs. The AlgEs comprise seven well-studied enzymes from Azotobacter vinelandii (AlgE1 to -7) (10, 12), PsmE from Pseudomonas syringae (13), and three recently identified Azotobacter chroococcum enzymes (AcAlgE1 to -3) (14). AlgEs consist of 1 to 2 catalytically active A-modules combined with 1 to 7 R-modules thought to modulate binding. Alginate lyases affect the viscosity of the polymer through depolymerization. They catalyze chain cleavage through β-elimination that generates an unsaturated 4-deoxy-l-*erythro*-hex-4-enepyranosyluronate (Δ) residue at the nonreducing end (15). Different lyases show various preferences for the four possible cleavage sites M↓M, M↓G, G↓M, and G↓G (16–18), and so lyase activity is inherently affected by epimerization patterns.

**FIG 1:**
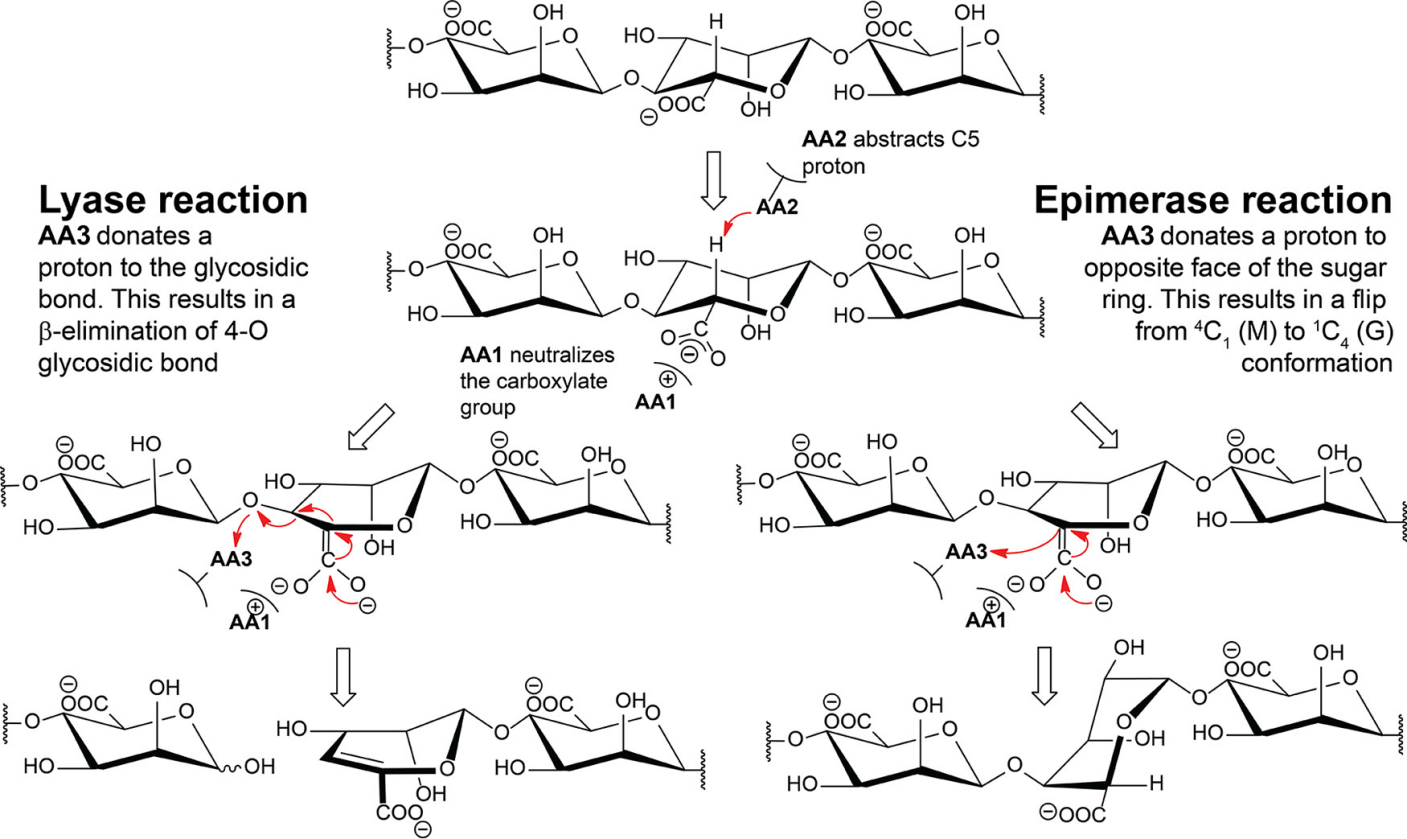
Proposed alginate lyase and epimerase reaction mechanisms. Both mechanisms start with proton abstraction from C-5 by AA2 (amino acid 2), requiring a charge neutralization of the carboxylate by AA1. In the lyase reaction, a proton is donated by AA3 to the leaving group of the glycosidic bond, whereas in the epimerase reaction, a proton is donated to the sugar ring to create the epimer. Only the M-lyase reaction is shown.

Mannuronan C-5-epimerases and lyases have been suggested to have similar reaction mechanisms (Fig. 1) (15), with common initial steps of charge neutralization by amino acid residue 1 (AA1) in Fig. 1 and proton abstraction from C-5 by AA2. The difference lies in the proton donation by AA3: in the epimerization reaction, it is given to C-5 from the opposite side from AA2 in the sugar ring, whereas in the lyase reaction, AA3 donates the proton to the O-4 oxygen involved in the glycosidic bond, and the polymer is cleaved. AlgE7, AcAlgE2, and AcAlgE3 display both activities, assumed from the same active site, highlighting the similarity between these mechanisms (16). All alginate epimerases share a YG(F/I)DPH(D/E) motif, ^149^YGFDPHE in the AlgEs (numbering according to AlgE7) (16, 19, 20). As the bifunctional AlgEs contain the same motif, other residues must also be involved in the cleavage reaction. This determinant residue(s) could work directly as the proton donor in the lyase reaction or indirectly by affecting a common AA3 to sometimes perform a cleavage event. The tyrosine (Y149) and the histidine (H154) in the motif are thought to be AA2 and AA3, although which role each residue performs is still not known (21, 22). For Pseudomonas aeruginosa AlgG, which has an active site similar to that of the AlgEs despite low overall sequence similarity, it has been proposed that water is AA3 and that histidine is AA2 (22). Acidic residues around the active site could have roles in modulating the pK_a_ values of active residues, or they could be involved in calcium binding. E155 might stabilize the substrate carboxylate group through the coordination of a calcium ion, together with D178, which is conserved in the AlgEs but not in the calcium-independent AlgG (21, 22). The calcium ion would then represent AA1. It is important to note that experiments in the absence of calcium cannot be used to probe if it actually has a catalytic role, as calcium is also required for the structural stability of the catalytically active A-modules and the carbohydrate binding module-like R-modules in the AlgEs (9, 21, 23).

There is evidence that the AlgEs form the distinct block patterns in alginate through a processive mode of action where every other sugar residue is epimerized (24, 25). Such an action would create MG blocks from a first binding event and G blocks from a second, explaining the occurrence of both block structures in alginate. Previous studies point to the importance of substrate interactions with charged residues far from the active site in activity and product patterns (21, 26–29).

In this study, we shed new light on the mode of action and catalytic mechanism of the bifunctional AlgE7 through investigations of the three-dimensional (3D) structure, through substitutions of amino acids in and around the active site, and by varying the reaction conditions (pH, [Ca], [NaCl], and substrates).

## RESULTS

### The lyase and epimerization activities are mutually dependent.

**FIG 2:**
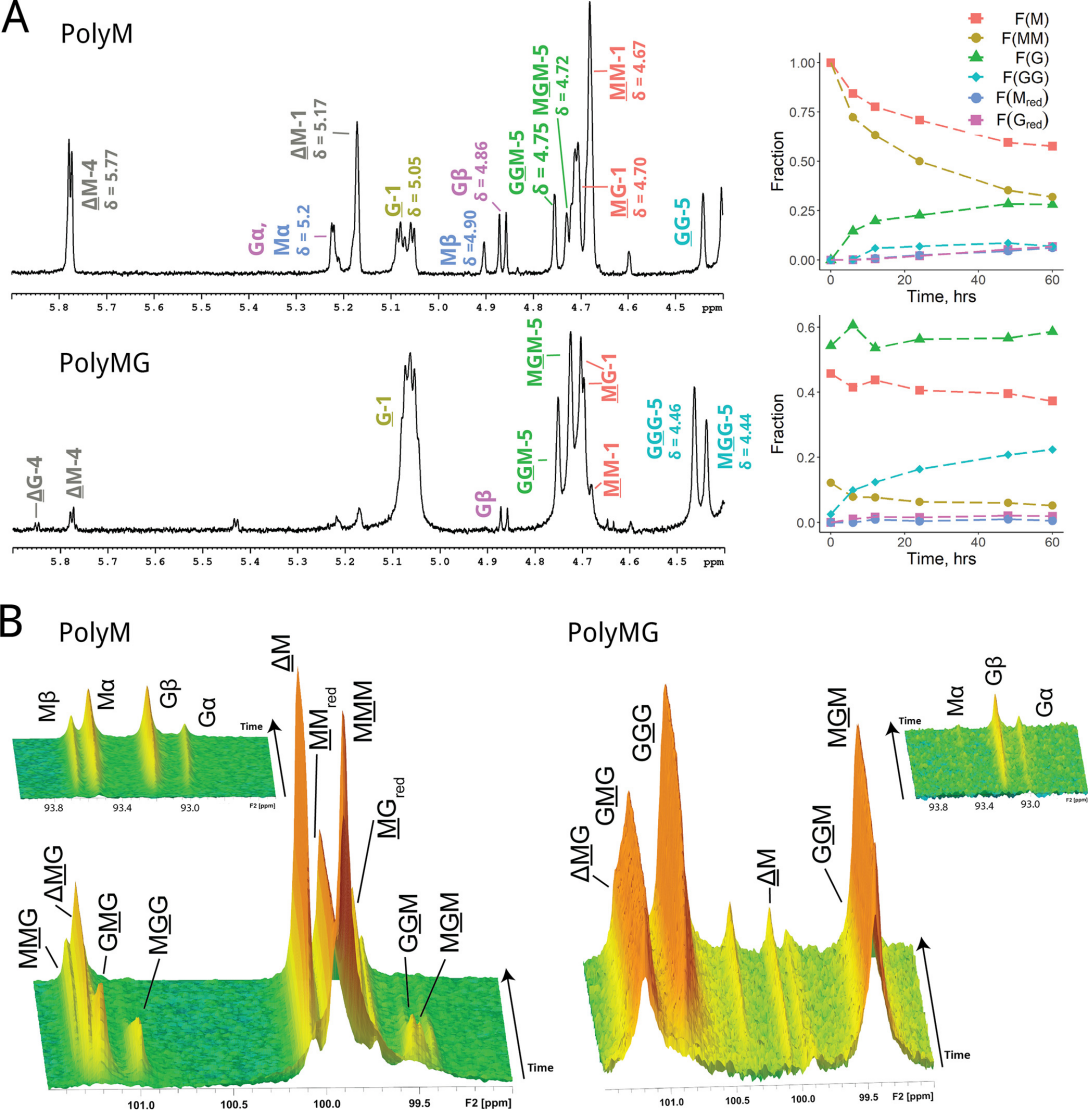
NMR spectra showing AlgE7 acting on polyM and polyMG. Underlined monomer residues give rise to the signals, and they give distinct peaks based on their nearest neighbor residues. (A) ^1^H spectra after epimerization for 60 h. The mixture contained 5 mM HEPES, 75 mM NaCl, and 2.5 mM CaCl_2_ (pH 7.0). A total of 2.5 mg/mL of the substrate and 92 nM enzyme were used in the reaction (1:300 [wt/wt] enzyme-to-substrate ratio). Fractions of residues calculated from the integration of the ^1^H spectra at different time points are plotted to the right and shown in Table S1 in the supplemental material (calculation of sequential parameters is explained in Fig. S3). (B) Time-resolved ^13^C spectra of the reaction from 0 to 1,000 min (16.7 h), recorded at 25°C, with a mixture containing 10 mM MOPS, 75 mM NaCl, and 2.5 mM CaCl_2_ at pH 6.9. A total of 11 mg/mL of the substrate and 2.9 μM enzyme were used (1:42 [wt/wt] enzyme-to-substrate ratio).

The epimerization pattern and preferred cleavage sites of AlgE7 have been studied previously (12, 16), and here, we further elucidate the mode of action by recording ^1^H NMR (nuclear magnetic resonance) spectra at different reaction time points and monitoring the reaction by time-resolved ^13^C NMR. End products were verified with ^13^C HSQC (heteronuclear single quantum coherence spectroscopy) spectra (Fig. 2; see also Fig. S1 and Table S1 in the supplemental material) and HPAEC-PAD (high-performance anion-exchange chromatography with pulsed amperometric detection). The investigation was performed using the two well-defined substrates polymannuronan (polyM) and polyalternating alginate (polyMG).

The ^1^H NMR spectrum of AlgE7 displayed in Fig. 2A on polyM shows an absence of ΔG signals and approximately equal amounts of M and G residues at reducing ends (M_red_ and G_red_, respectively). After an initial buildup, there is a decline in G residues, seen by the removal of MGG and GGM (underlining indicates the residue in question) (Fig. 2B) during the reaction course, implying that AlgE7 is able to abstract a proton from G residues to create Δ residues. Moreover, from the time-resolved ^13^C NMR spectra (Fig. 2B), we can infer that AlgE7 is also able to catalyze the lyase reaction (i.e., donate a proton to the 4-O) within an M block, as it creates ΔM from the start. This corresponds to the potential cleavage sites G↓MM, G↓GM, M↓GM, and M↓MM (the arrow denotes cleavage of the 4-O glycosidic bond). The number of G residues in the substrate increases during the reaction, but it is seen from the integrals of the ^1^H spectra in Fig. 2A that the formation rates of both M and G reducing ends remain similar for the duration of the reaction. The fact that the cleavage rates for G↓XM and M↓XM remain similar despite their relative concentrations changing drastically implies that cleavage happens in the same binding event as preceding epimerizations. The ΔM peak is much larger than the ΔMG peak, suggesting that mostly ΔMM is created, and this is supported by the HSQC spectrum recorded at the end of the reaction (Fig. S1). This is also consistent with a cleavage event happening after processive epimerizations, with a polyM block ahead. Since we see GG signals in the proton spectrum after 60 h, XGGMX motifs could have become cleavage sites forming XGG_red_ and ΔX. No GGG signals were present in any of the spectra on polyM, indicating that there was no formation of G blocks. The absence of consecutive G-residue signals in the HSQC spectrum at the end of the reaction supports the cleavage of G residues.

When polyMG was used as the substrate, the pattern changed. The only possible cleavage sites are G↓MG, M↓GM, G↓GM, and M↓GG, as only negligible amounts of consecutive M residues are present. The ^1^H and time-resolved spectra show that the lyase activity decreased, only a small ΔG signal appeared, and almost no M_red_ was formed. This finding corresponds to weak activity on the cleavage site G↓XG, which was not found on polyM, and implies a clear preference for cleaving G↓GM. From the time-resolved spectra, we see that G-block-rich alginate is produced, as the MG blocks are filled in. A total lack of activity was found on pure oligo-G, excluding cleavage inside G blocks (Fig. S2). In conclusion, the degree to which the substrate is epimerized appears to be highly correlated with the lyase activity.

The spectra shown in Fig. 2A and B were recorded with 75 mM NaCl to reduce gel formation, but ^1^H NMR spectra recorded without NaCl gave a different product pattern. This was most apparent on polyMG, where NaCl was seen to enhance epimerization and reduce lyase activity (Fig. S4 and Table S1). ^13^C NMR experiments on polyM without NaCl showed the same trends as those with 75 mM NaCl except for a total lack of MGG signals during the reaction course (results not shown). The effect of reaction conditions was investigated in more detail and is described below.

HPAEC-PAD chromatograms of the samples epimerized for 60 h in the presence of NaCl (Fig. S5) showed that AlgE7 displays a pattern that is different from those of the M- and G-specific lyases acting on similar substrates. This reflects the more complex product patterns caused by the bifunctional enzyme. The peaks corresponding to the products created by AlgE7 overlap approximately those of 3- to 6-mers created by an M-specific lyase on polyM and polyMG, whereas the peaks do not overlap the pure G oligomers created by the G-specific lyase on polyG.

### Molecular basis for the lyase activity of the bifunctional enzymes and identification of R148 as a central residue.

**FIG 3:**
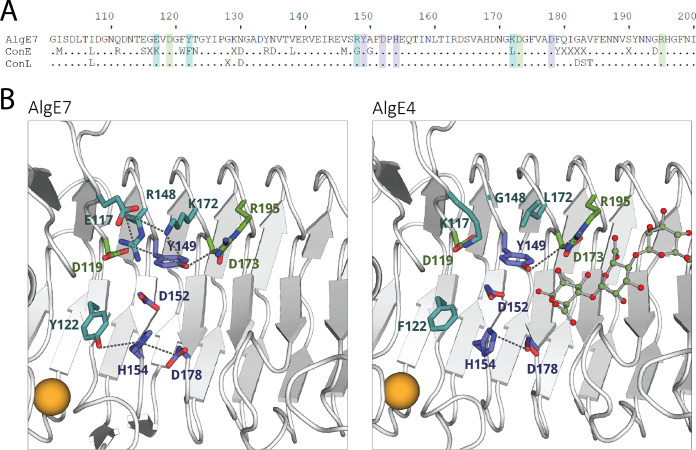
(A) Alignment of AlgE7 from A. vinelandii with two consensus sequences. ConE is the consensus sequence of the A-modules displaying only epimerase activity from A. vinelandii and *A. chroococcum* (AlgE1A1, AlgE1A2, AlgE2A, AlgE3A1, AlgE3A2, AlgE4, AlgE5, AlgE6, and AcAlgE1). ConL is the consensus of the A-modules that have both lyase and epimerase activities (AlgE7, AcAlgE2A, and AcAlgE3A). Residues that are not conserved in the consensus sequence are represented in the alignment as “X,” whereas dots denote residues that are almost or completely conserved. Highlighted letters indicate amino acids replaced in this study: purple indicates the catalytic amino acids, blue indicates the four residues differing between ConE and ConL, and green indicates other studied residues. AlgE7 has 82% identity with ConL and 63% identity with ConE. The alignment and consensus sequences are made from global protein alignments using the BLOSUM 62 scoring matrix. (B) The residues highlighted in panel A, shown in sticks in the crystal structure of AlgE4 (right) (PDB accession number 2PYH), and a homology model of AlgE7 (left), with the same color code as the highlights in panel A. Dashes are shown between residues that potentially form hydrogen bonds. The calcium ion needed for structural stability is visualized as an orange sphere. The model of the AlgE7 A-module was built by SWISS-MODEL (30), using the structure of the AlgE4 A-module (PDB accession number 2PYH), and then energy minimized using the YASARA server (31).

To understand the dualistic activity of AlgE7, we wanted to see how the ratio between the two activities is affected by specific amino acid substitutions. We assumed that the same active site is responsible for both lyase and epimerase activities, containing the four catalytic residues Y149, H154, D152, and D178 (16, 21). Previously, a bifunctional enzyme was created, consisting of the N-terminal part of AlgE7 and the C-terminal part of AlgE1 (16). The mutational study therefore focused on the N-terminal residues in the binding groove (from amino acid residues G100 to I200), as these appeared to be sufficient for lyase activity. Targeted residues are highlighted in the sequence alignment comparing the consensus sequences of the 3 bifunctional A-modules (ConL) and the 10 epimerase A-modules from A. vinelandii and A. chroococcum (ConE) (Fig. 3A; Fig. S6). A homology model of AlgE7 was generated using the most closely related available structure of AlgE4 (21). The model was built with SWISS-MODEL (30) based on the crystal structure of AlgE4 (PDB accession number 2PYH) and then energy minimized using the YASARA server (31). Charged and polar residues take part in an extensive hydrogen-bonding network formed in the binding groove, illustrated with dashes in Fig. 3B. Specifically, residues 117, 122, 148, and 172 show differences in polar characteristics that appear to affect the positioning of the active-site residues Y149 and H154 (Fig. 3). Hydrogen-bonding networks have previously been shown to be important for the binding and processive action of epimerases (28).

**FIG 4:**
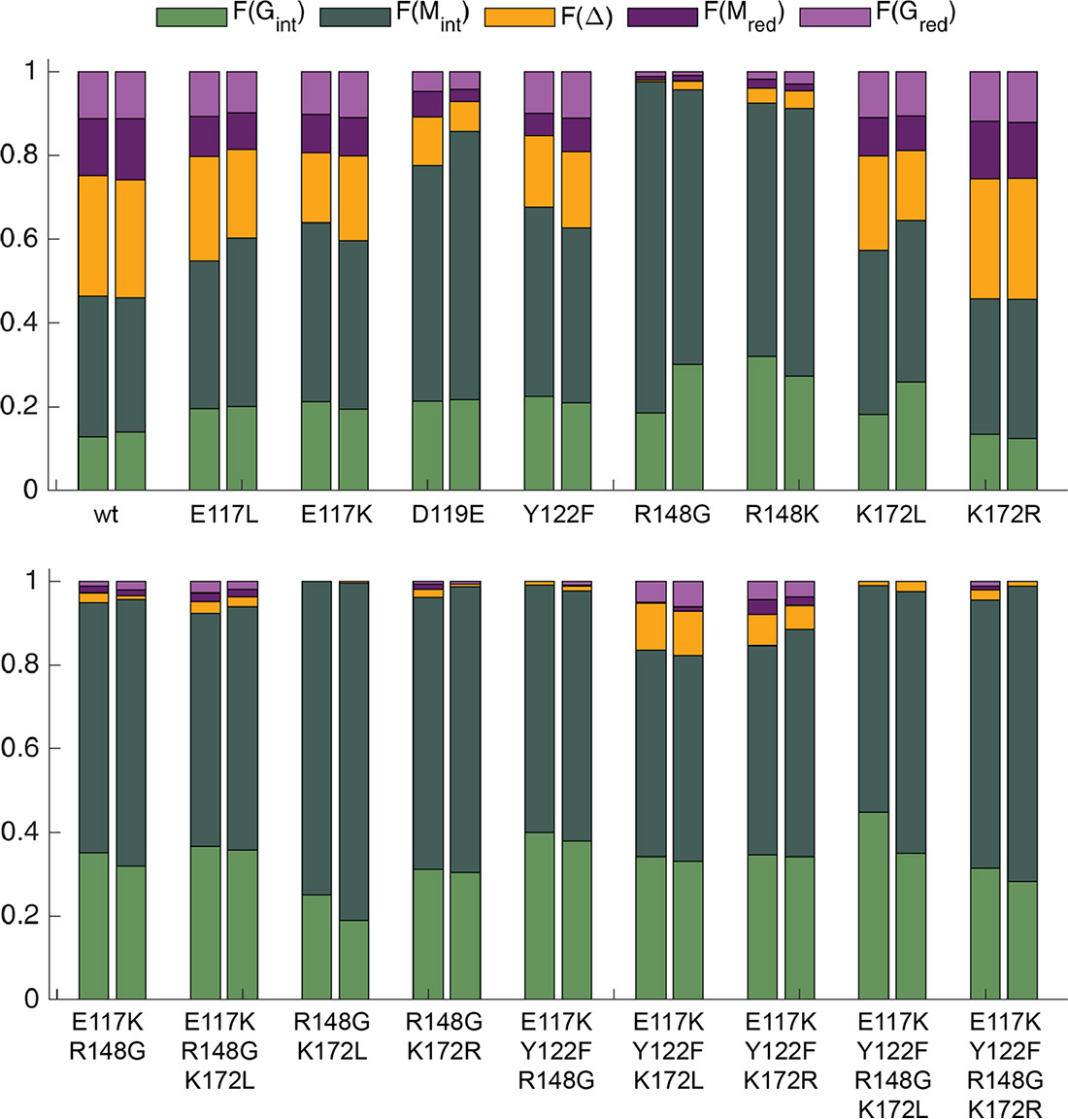
Product profiles from endpoint ^1^H NMR of wild-type (wt) and mutant AlgE7 proteins acting on 1 mg/mL polyM, with 2.5 mM CaCl_2_ added to the reaction mixtures, incubated at 25°C for 24 h. A total of 40 μL of enzyme extracts was added to 500 μL of the reaction mixture. Each column represents one experiment, and duplicates are positioned together. Green, internal G residues; orange, Δ residues; dark green, internal M residues; dark purple, M residues at reducing ends; light purple, G residues at reducing ends. The sum of these fractions amounts to 1.

Thirty-three variants of AlgE7 with single amino acid substitutions and combinations of these were created based on sequential and visual analyses as illustrated in Fig. 3. The following paragraphs describe each group of created variants in more detail. The activities of the variants with polyM were measured by three different methods. First, the release of ^3^H from 5-^3^H-labeled mannuronan was detected in a ^3^H assay (32, 33). This assay measures directly the first step (proton abstraction) of the reaction (Fig. 1). As this step is common for both activities, the ^3^H assay measures the total enzyme activity. Second, lyase activity was measured by detecting the increase in the absorbance at 230 nm upon the generation of the unsaturated double bonds in the resulting Δ residues (Fig. 1). The initial slope of the reaction was compared to that of the wild type. Results from the first two activity assays are shown in Table 1. Third, product profiles after a 48-h reaction of 22 of the variants were analyzed by ^1^H NMR. Results from 18 of these variants are shown in Fig. 4, whereas the D119A, D119N, Y122A, and D173A variants had no detectable activity and are excluded from the figure (values are listed in Table S2). These experiments were performed using partially purified enzyme extracts where the exact enzyme concentrations cannot be determined. Therefore, the results in Table 1 and Fig. 4 describe qualitative differences between the variants.

**TABLE 1:**
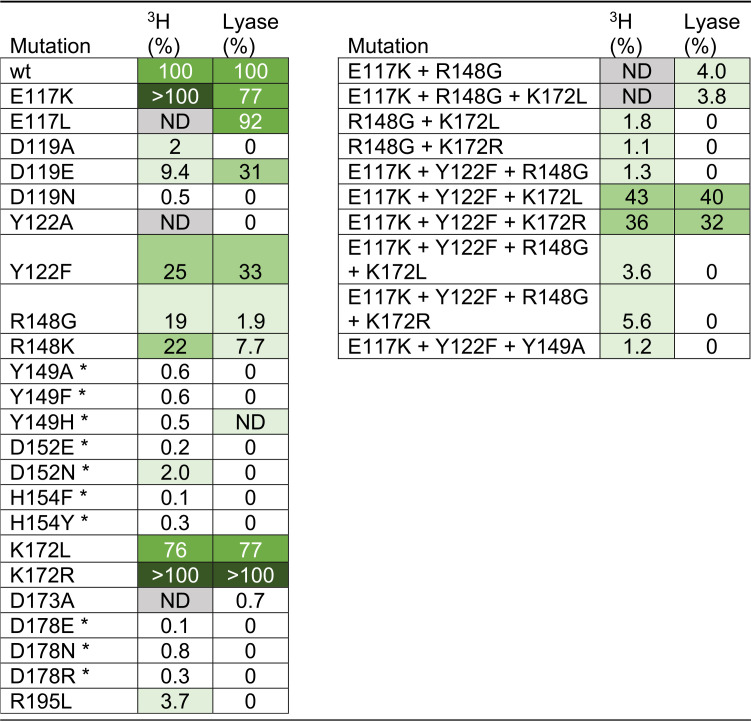
Results from ^3^H assays and lyase assays of AlgE7 variants on polyM^*a*^

a All results are percentages of the wild type (wt). Bright green indicates activity between 60 and 100% of that of wild type, dark green is activity above 100% of that wild type, light green is between 20 and 59% of that of wild type, and pale green is between 1 and 19% of that of the wild type. An asterisk denotes residues involved in catalysis. ND (not determined) indicates that the experiment has not been performed. In the ^3^H assay, 0.25 mg/mL of 5-^3^H-labeled polyM was incubated with 2 mM CaCl_2_ and enough enzyme extract (from 5 to 100 μL in a 0.6-mL total volume) to give comparable signals. In the lyase assay, 20 μL of enzyme extracts was added to a total of 250 μL of reaction buffer containing 2.5 mM CaCl_2_ and 1 mg/mL polyM.

Residues E117, Y122, R148, and K172 are all invariantly conserved in the bifunctional enzymes, whereas at the corresponding positions, K117, F122, G148, and L172 are conserved in the epimerases. In the epimerases, K117 forms a salt bridge with the conserved residue D119 (Fig. 3). In the bifunctional enzymes, D119 is instead interacting with R148. R148 and K172 introduce additional positive charges close to the active-site residue Y149. Y/F122 is the only one of the four residues situated close to the catalytic histidine. We expected to suppress the lyase activity by exchanging all four residues and combinations of them into the corresponding residues found in the epimerases (E117K, Y122F, R148G, and K172L), and we wanted to see which of these residues exerted the largest effect on the activity. The variants E117L, Y122A, R148K, and K172R were created to investigate the role of the side chains further, and K172R was also included in the combination variants.

**FIG 5:**
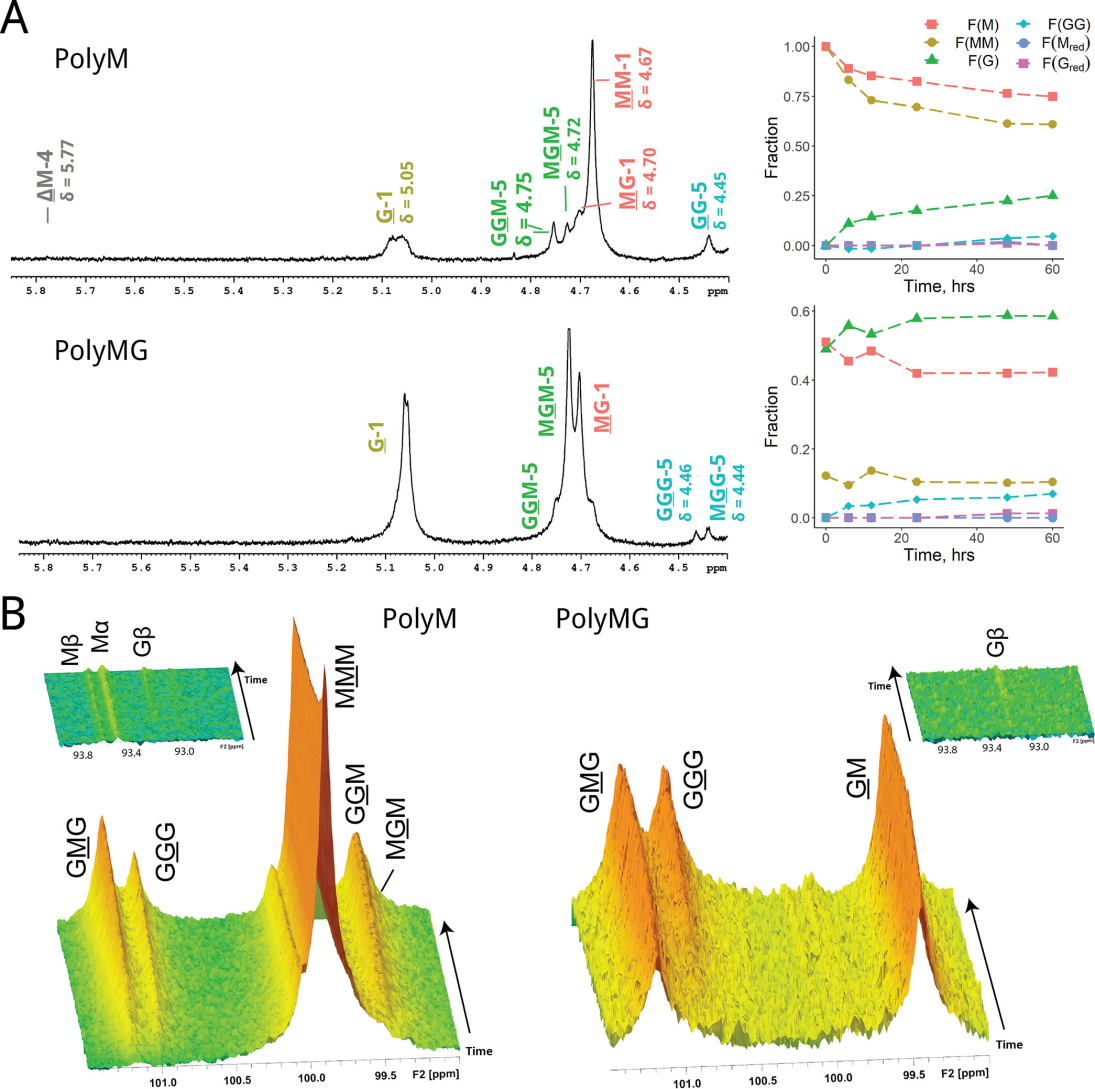
NMR spectra showing R148G activity on polyM and polyMG. Underlined monomer residues give rise to the signals, and they give distinct peaks based on their nearest neighbor residues. (A) ^1^H spectra after epimerization for 60 h, in a mixture containing 5 mM HEPES, 75 mM NaCl, and 2.5 mM CaCl_2_ (pH 7.0). A total of 2.5 mg/mL of the substrate and 0.092 μM enzyme were added (1:300 [wt/wt] enzyme-to-substrate ratio). Plots show fractions calculated from the integration of spectra recorded at different time points of the reactions. (B) Time-resolved ^13^C spectra from 0 to 1,000 min (16.7 h), recorded at 25°C with a mixture containing 10 mM MOPS, 75 mM NaCl, and 2.5 mM CaCl_2_ at pH 7.0. A total of 11 mg/mL of the substrate and 3.0 μM enzyme were used (1:42 [wt/wt] enzyme-to-substrate ratio).

The most interesting single variant turned out to be R148G, which almost completely abolished the lyase activity while preserving the epimerase activity, both alone and in combination variants (Table 1 and Fig. 4). The variant R148K retained reduced yet significant lyase activity. Because of its significance for lyase activity, we purified the R148G variant and performed the same ^1^H NMR, time-resolved ^13^C NMR, and HSQC experiments for detailed characterization as the ones described above for the wild type, on both polyM and polyMG (Fig. 5; Fig. S1 to S4 and Table S1). From the plot of ^1^H NMR integrals on polyM, we see that R148G produces G residues and GG dyads at a lower rate than the wild type. It also forms GGG motifs that were not observed with the wild-type enzyme. On polyMG, it creates GGG signals only when NaCl is present (Fig. 5; Fig. S4). Also, for the wild type, G-block production is increased when NaCl is present (Fig. 2).

In contrast, E117K/L and K172L/R have lyase activity (Table 1) and product profiles (Fig. 4; Table S2) similar to those of the wild type. Y122F has reduced activity measured by the assays in Table 1 but displays a similar product profile at reaction completion. Y122A, on the other hand, was inactive. When combining E117K, Y122F, and K172L/R, the activity assays were similar to that of R148G/K, whereas the product profile showed higher lyase and epimerase activities. This points to indirect roles of these three residues in catalysis, as their replacements are not detrimental to the lyase activity. Nevertheless, E117L/K, Y122F, and K172L appear to be connected to substrate specificity, as they produce larger amounts of G_red_ than M_red_, as opposed to the wild type, which produces slightly more M_red_. The variant E117K+Y122F+K172L produces almost exclusively G_red_.

D119, D173, and R195 are conserved residues in a hydrogen-bonding network around the catalytic residue Y149. The roles of these residues were investigated using the variants D119A, D119N, D119E, D173A, and R195L. D119E retained 9% of the total activity and 31% of the lyase activity, whereas D119A and D119N retained only 0.5 to 2% of the total activities (Table 1), implying that the charge of the side chain is more important than its size. Of the three variants, only D119E had detectable activity in the ^1^H NMR experiment. It produced more G residues than the wild type, which could be a result of its lower lyase activity (Fig. 4). As seen in Table 1, the AlgE7 D173A variant displayed measurable lyase activity, but it was too low to be detected in the ^1^H NMR experiments. R195L had no detectable lyase activity and displayed low total activity in the ^3^H assay but was not investigated by ^1^H NMR.

To investigate the roles of the proposed catalytic acid and base in the reaction, we created the active-site variants Y149A, Y149F, Y149H, H154F, and H154Y. All three variants at position 149 had a residual activity of around 0.5% of that of the wild type (Table 1). H154Y displayed a similar level of residual activity, whereas the activity of H154F was close to the detection limit arising from the noise. The other two proposed active-site residues D152 and D178 were also investigated, using variants D152E, D152N, D178E, D178N, and D178R. Both D152N and D178N display higher residual activity than D152E and D178E, implying that for these aspartates, size is more important than their negative charge. The D178R variant was inactive, which is not unexpected for such a dramatic change in the active site. ^1^H NMR experiments were not performed for any of these variants.

### Complex effects on the dual activities are observed by varying the reaction conditions.

**FIG 6:**
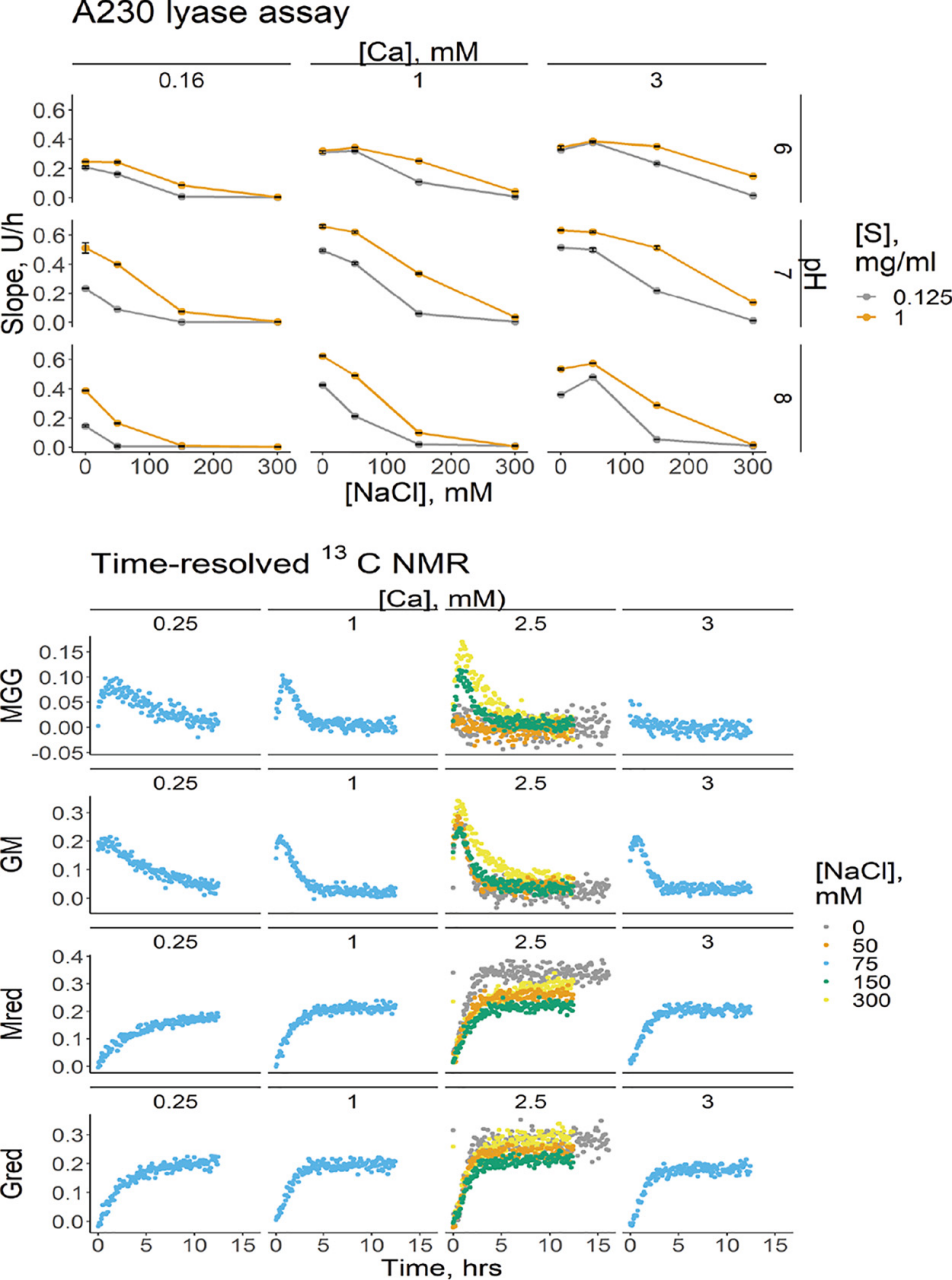
Salt dependency experiments of wild-type AlgE7. (Top) Slopes from a factorial spectrophotometric lyase activity assay at two different substrate concentrations [S], three different pHs, three different calcium concentrations, and four different NaCl concentrations. A total of 0.3 μM enzyme was added to each reaction mixture. The slope is given in units per hour, where U is the absorbance units at 230 nm. Points are averages from two repetitions, and error bars show the ranges of values for the repetitions. (Bottom) Integrated time points from ^13^C NMR with a constant substrate concentration (9 mg/mL) and constant pH (6.9) at four different calcium and salt concentrations. A total of 3 μM enzyme was added to each reaction mixture. *y* axes show the relative integrals of peaks representing the given residues.

As mentioned above, the addition of NaCl boosted the G-block production of both the wild type and R148G, especially on polyMG (Fig. 5; Fig. S4). In addition to the effect of NaCl on lyase activity, we also investigated the calcium concentration, pH, and substrate concentration in more detail. The lyase activity of AlgE7 was monitored in a factorial experiment where combinations of added NaCl (0, 50, 150, and 300 mM), calcium chloride (0, 1, and 3 mM), pH (6, 7, and 8), and the substrate (1 and 0.125 mg/mL) were tested. Calcium is required in the enzyme buffer to maintain the structural stability of the enzymes, and a minimum of 0.16 mM calcium was present in all reaction mixtures. Lyase activity was measured with a spectrophotometric assay to detect the increase in the absorbance at 230 nm upon the creation of Δ residues (shown in Fig. S7), and the initial slope (Fig. 6) and endpoint values (Fig. S8) were calculated (Table S3).

High calcium concentrations decreased the negative effect of NaCl on lyase activity, and at 3 mM calcium, the activity at 50 mM NaCl was comparable to the activity at 0 mM NaCl without supplemented calcium. The sensitivity to NaCl was also decreased with decreasing pH, although the optimal pH in general is 7. Positively charged residues are involved in the substrate interaction as well as the lyase reaction through R148 and K172, and pH could affect the protonation state of these residues. Activity increased with the substrate concentration, except for pH 6, 0 mM NaCl, and 1 to 3 mM CaCl_2_, where the activity was the same at 0.125 mg/mL of the substrate as that at 1 mg/mL. Similar trends were otherwise observed at the two substrate concentrations. The enzyme kinetics cannot be modeled by classical Michaelis-Menten kinetics due to the continuously changing substrate from cleavage and epimerization.

To analyze the lyase and epimerase activities simultaneously, different NaCl and calcium concentrations at pH 7 with 9 mg/mL of polyM were tested by time-resolved ^13^C NMR (Fig. 6, bottom). In the experiments where the NaCl concentrations were changed, the calcium concentration was kept constant at 2.5 mM. The NMR experiment was performed at higher substrate concentrations than those for the lyase assay in order to obtain a sufficient signal-to-noise ratio. One observed difference that could be caused by this is that the lowest lyase activity was seen at 150 mM NaCl, unlike in the lyase assay, where the lowest activity was seen at 300 mM NaCl. Both the epimerase and lyase reactions increased with the calcium concentrations. However, calcium inhibited the formation of consecutive G residues. At high calcium concentrations (2.5 and 3 mM) and at NaCl concentrations below 150 mM, almost no MGG triads were produced, unlike at higher NaCl or lower calcium concentrations, where MGG signals were produced initially and then removed through cleavage. MG and GG formation were highest at 300 mM NaCl, but this was not explained by a decrease in lyase activity.

## DISCUSSION

### Mode of action of wild-type AlgE7.

In the first experiments presented, we observed that the lyase and epimerase activities are affected by the type of substrate, resulting in different product patterns. This is a challenging system for experimental investigation as the lyase activity is affected by epimerization, and the degradation of epimerized residues hinders the measurement of the actual epimerization activity. When A. vinelandii secretes the alginate used in its protective coat, the alginate has already gone through modifications by the periplasmic epimerase. AlgE7 and the other extracellular epimerases and lyases therefore encounter a substrate that is not pure polyM. The extracellular enzymes take part in forming the final alginate product in even more complex ways than what we illustrate here with AlgE7 alone, and AlgE7 itself is not expressed at large levels compared to some of the other epimerases (34). The biological role of AlgE7 has previously been indicated by the release of alginate from the cell surface of A. vinelandii after secretion, as *algE7* knockout mutants do not release alginate into the medium (35). Newly secreted alginate contains M residues with occasional G residues stemming from the activity of periplasmic AlgG, and we indeed see the highest activity of AlgE7 on polyM compared to polyMG.

**FIG 7:**
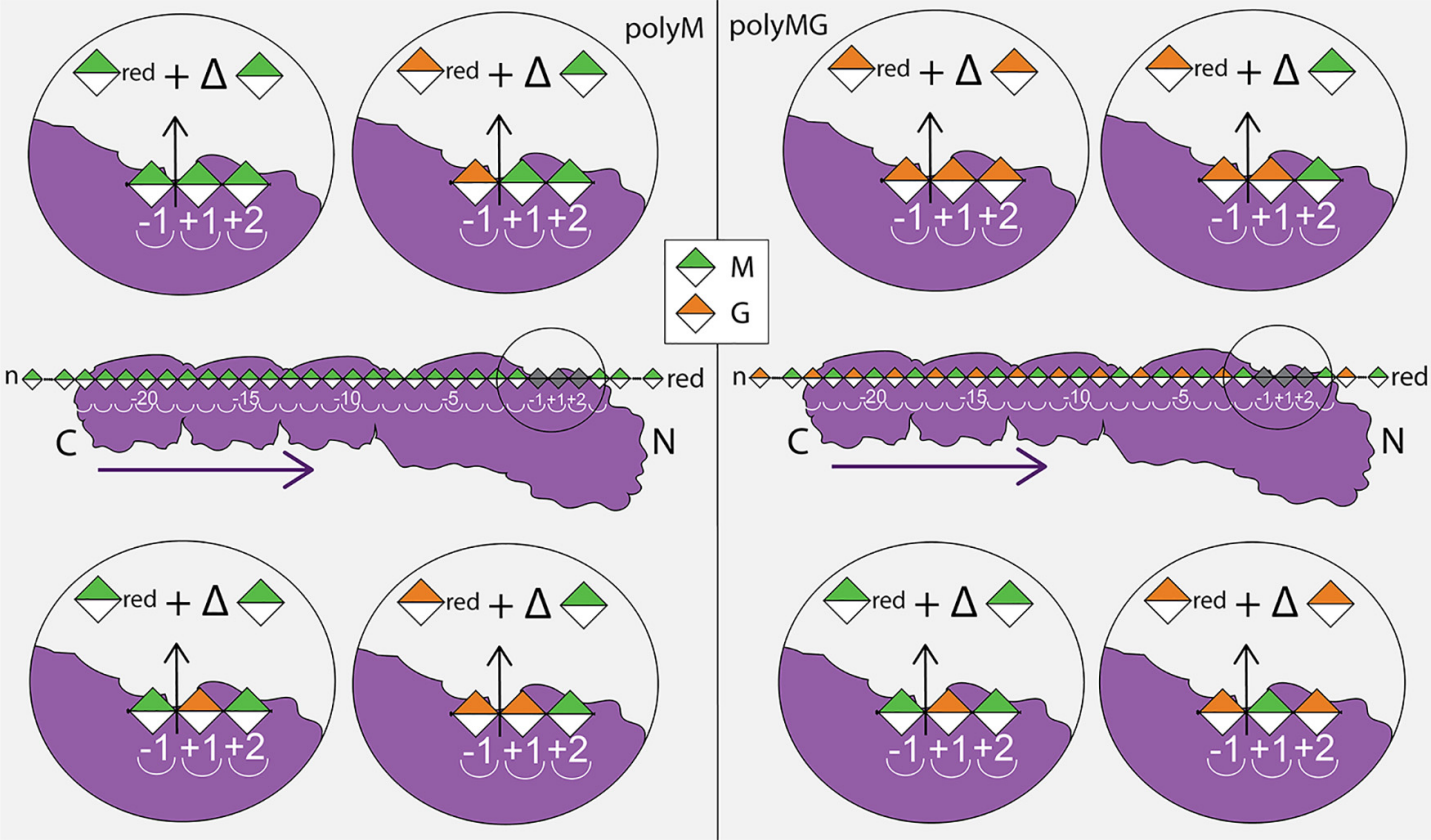
Illustration of the possible cleavage sites from the observed product patterns of AlgE7 on polyM (left) and polyMG (right). In the middle, AlgE7 is shown as it binds to the substrate that it is presented with initially, but this will change during the reaction course. Except for M↓MM, all cleavage sites are in accordance with cleavage after a processive binding event or due to a preferred attack of the end of MG and GG sequences. For polyM, we observed the creation of M_red_, G_red_, and ΔM, which can be caused by cleavage at the four cleavage sites illustrated in the left panels. On polyMG, mainly G_red_ and ΔM are observed, corresponding to cleavage site G↓GM. However, we also observe small amounts of M_red_ and ΔG, and four cleavage sites shown in the right panels are possible on polyMG. Sugar residues are colored according to the symbol nomenclature for graphical representations of glycans (49). The purple arrows indicate the proposed direction of movement for the processive AlgE4 (28). AlgE7 consists of one A-module and three R-modules, and the outline of all four modules is shown. The spatial arrangement of these modules is suggested to be elongated, but this has not been studied experimentally.

When M-rich alginate is the initial substrate, the potential cleavage sites are G↓XM and M↓XM (Fig. 2), whereas on polyMG, G↓GM and G↓XG are indicated. Evidence is found that AlgE7 can cleave before both M residues and G residues, which gives the eight potential cleavage sites shown in Fig. 7. AlgE7 has much higher lyase activity when acting on polyM than when acting on polyMG, and it cannot cleave the pure G substrate. It does not create more than two consecutive G residues on polyM before they are removed through cleavage events. To explain these findings, we hypothesize a mode of action where cleavage represents the last step of processive epimerization, before product dissociation and release (Fig. 7). Alternatively, preferred attack at the reducing end of MG blocks flanking M blocks and G blocks flanking MG blocks would give the same results. Both hypotheses explain why we see approximately equal amounts of M_red_ and G_red_ produced during the whole reaction course on polyM, even though the concentration of G residues increases. On polyMG, on the other hand, we observe mainly G reducing ends and almost no M reducing ends, caused by cleavage after the formation of G blocks. These hypotheses also explain why we observe ΔG signals only from the MG substrate, because assuming that AlgE7 moves relative to its substrate in the same direction as AlgE4 (28), it could create those signals on polyM only in the unlikely event that it reaches the end of another MG block. More ΔMM is produced than ΔMG from polyM (Fig. 2B), and on M-rich alginate, attacking the reducing end of G and MG blocks would likely produce ΔMM. The fact that the enzyme cannot cleave pure G-alginate also fits into this hypothesis, as this alginate cannot be epimerized. The exact positioning of various substrates might determine if epimerization or cleavage happens, which could be determined by substrate features. MM residues are more outstretched in the chain than alternating MG or GG residues and span around 10.5 Å, compared to around 8.7 Å for GG residues (36). In addition, M residues have both a hydrophilic face and a hydrophobic face, whereas G residues lack a hydrophobic face. They also have a different configuration of all the polar groups around the ring carbons, and due to their distinct chair conformations (^4^C_1_ for M and ^1^C_4_ for G), they result in different three-dimensional structures of the alginate chain.

### The enzymatic mechanism of epimerization and cleavage.

**FIG 8:**
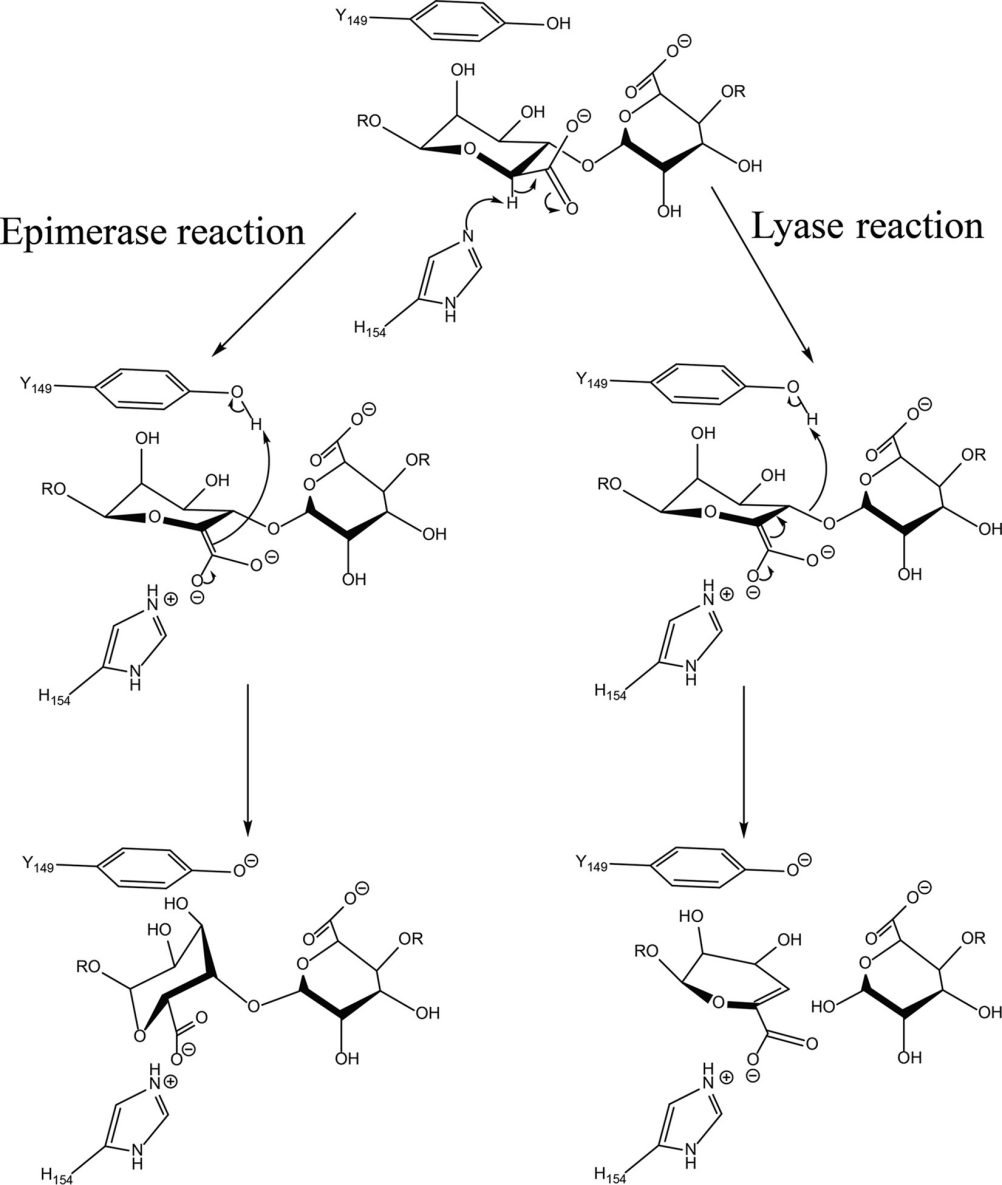
Proposed mechanisms of the epimerization and lyase reaction of AlgE7 on M residues (XMMX substrates).

The substrate might affect whether epimerization or cleavage happens, but the reason why only some of the AlgEs are bifunctional is due to sequence determinants in the enzymes. As three of the four important determinants (E117, R148, and K172) are positioned on the side of the active-site tyrosine Y149, we propose that H154 is the proton abstractor common in both reactions (AA2 in Fig. 1) and that Y149 is the catalytic acid (AA3) (Fig. 8). The orientation of a ligand observed in the crystal structure of AlgE4 is with the reducing end toward the N terminus of the protein, and this has also been deemed the most likely orientation during processive action through molecular dynamics simulations (21, 28). This orientation seemingly rules out that Y149 could abstract the H-5 protons from M residues, as illustrated in Fig. 8. In Fig. 8, a sugar residue is modeled in the active site based on an elongation of the crystal structure ligand, placing the H-5 in an angle that is accessible for H154 but not for Y149. After the abstraction of H-5, on the other hand, the opposite plane of the sugar is available for proton donation by Y149. The exact positioning of Y149 relative to the substrate could govern its donation to either C-5 or O-4 of the residue in subsite +1. This illustrates how epimerization at C-5 gives rise to significant differences in how the ligand is placed in the binding site. We therefore cannot be sure if the same residue performs proton abstraction of both M residues and G residues, as the positioning would be different. Alginate lyases that can cleave G residues can perform an anti-β-elimination using either a histidine or a tyrosine as a base and a tyrosine as an acid (37, 38). Based on this, it cannot be ruled out that Y149 might perform the role of the base when the enzyme removes G residues, as it might be in a better position for this than the histidine (seen in the last step of epimerization in Fig. 8). The two active-site aspartates D152 and D178 have been proposed to be associated with substrate stabilization in AlgE4 (21), and D178 could also neutralize the charge of the carboxylate group through the coordination of a calcium ion (22). For simplicity, we do not show the neutralization of the carboxylate charge in Fig. 8.

R148 was shown to be the most important residue for the lyase activity of AlgE7, as the lyase activity almost completely disappeared in the R148G variant. Based on the result that the R148K variant retains slightly more lyase activity than R148G, the charge of R148 seems to be important, whereas the guanidino group of the arginine is required for optimal activity (Table 1 and Fig. 4). We have considered the possibility that R148 donates the proton to the glycosidic bond itself, but it is located too far from the glycosidic bond in the proposed reaction mechanism (Fig. 3 and 8). We propose that R148 is essential in influencing whether Y149 donates the proton to the other face of the sugar ring (epimerization) or to the glycosidic bond (lyase reaction) and that without it, only epimerization happens.

The role of E117, R148, and K172 might be to position R148, Y149, and the substrate residue in the active site through electrostatic interactions as well as perturbing the pK_a_ of groups involved in catalysis. Even when combining the E117K, Y122F, R148G, and K172L substitutions, a minute lyase activity can be observed in the NMR spectra (Fig. 4), suggesting that these residues are involved in the lyase reaction only indirectly. The effects of the variants E117K, Y122F, and K172L on the lyase activity were not as large as those for R148. However, they produced more G residues than M residues at reducing ends, unlike the wild type, which produced similar amounts. The variants therefore prefer G↓MM over M↓XM. This result highlights the sensitive fine-tuning of interacting residues relative to the positioning of the specific substrate in catalysis. E117 and K172 are both situated slightly away from the putative substrate binding site and likely affect substrate binding indirectly through the hydrogen-bonding network that includes R148 and Y149 (Fig. 3). As Y122A was inactive, the aromaticity and/or size of this residue is essential. Similar results were obtained for AlgE4, where the F122Y variant retained 65% of the wild-type activity, whereas the F122V variant retained only 0.2% (21). AlgG has a tyrosine positioned in the corresponding position, and although the alanine variant of this residue was not detrimental, it reduced the activity to 20% of the wild-type activity, compared to 66% for the phenylalanine variant (22). For AlgG, it was concluded that this residue has a role in coordinating the active-site histidine. We also propose that it has a role in the correct positioning of the substrate for lyase activity.

The hydrogen-bonding network between the charged residues around the active site is important not only for the catalytic mechanism but also for the processive mode of action of the AlgE epimerases. D119A, D173, and R195 are conserved in the block-forming AlgE epimerases but have no equivalents in AlgG, which introduces only single G residues to alginate chains. Substitutions of these residues that remove the charges are detrimental to or at least diminish activity dramatically, as seen here for AlgE7 (Table 1) and previously for AlgE4 (21, 28). Block formation requires either processive action with the ability to stay attached to the substrate for consecutive catalytic events or at least a preferred attack with the ability to bind specifically to already-epimerized alginate chains. The strength of the substrate interaction would be essential in both scenarios and could be the property that is affected when D119, D173, and R195 are mutated.

High NaCl concentrations inhibited the initial lyase activity in the spectrophotometric lyase assay but not in the time-resolved NMR experiments (Fig. 6), which could be explained by the higher substrate concentrations of the latter (9 mg/mL, against 0.125 to 1 mg/mL in the lyase assay). Na^+^ and Cl^−^ ions shield charged residues in the substrate and enzyme that are essential for substrate interactions. A high substrate concentration would reduce the requirement for a strong enzyme-substrate interaction, as new substrate chains would be easily available for binding. Charged residues (E117, R148, and K172) are also implicated in the catalytic reaction, but if the shielding of these were detrimental, we would not expect that a high substrate concentration would diminish the effect from NaCl. The loss of G-block formation at high calcium concentrations, but only at low salt concentrations, probably stems from sodium ions shielding the charges in the MG substrate, preventing it from forming junction zones with calcium that would render it inaccessible to subsequent G-block formation. We see that NaCl promotes the formation of G blocks for both the wild type and R148G (Fig. 2 and 5; see also Fig. S4 in the supplemental material), and as the same result is seen for the lyase-inactive variant, it must be caused by an increase in epimerization activity and not only a change in lyase specificity. At the low substrate concentrations in the lyase assay, this effect would be smaller, and here, calcium is instead seen to save the activity at high NaCl concentrations. It would be interesting to further investigate the effects of different substrate concentrations, in combination with an investigation of residue or module determinants of the substrate interaction and processive mode of action.

### Conclusions.

In summary, the results presented here demonstrate what was previously suggested, namely, that the epimerization and lyase activities of the bifunctional AlgE7 share the same catalytic site. The two activities affect each other by constantly changing the alginate chains, and product formation is highly dependent on which substrate the enzyme is acting on. The lyase preferential cleavage sites are found to be M↓XM and G↓XM when using the well-defined alginate substrates polyM and polyMG. The mechanistic insights obtained through amino acid replacements and structural analysis suggest a likely catalytic reaction mechanism on M residues, where H154 acts as a catalytic base (AA2) in the reaction and Y149 takes the role as a catalytic acid (AA3). The variant R148G, positioned next to the catalytic residue Y149, abolished almost all lyase activity but retained epimerization activity and is thought to affect whether Y149 donates the proton to the glycosidic bond or to the other side of the sugar ring. Cleavage of both M and G residues is evident from the identified reaction products. The reaction conditions affect the enzymatic activity, where higher NaCl concentrations reduce lyase activity and increase G-block formation, whereas high calcium concentrations increase lyase activity and decrease G-block formation. The findings in this study explain the mechanistic basis behind the dual activity of AlgE7, in comparison to the AlgEs with mainly epimerase activity. New insight into how the two activities can be modulated relative to each other, in terms of both amino acid residues and reaction conditions, will aid in future applications of the AlgEs for the tailoring of alginates.

## MATERIALS AND METHODS

### Alginate substrates.

All alginate substrates were freeze-dried and weighed out upon use. Polymannuronan (polyM) was produced from the epimerase-deficient AlgG of Pseudomonas fluorescens strain PF20118 (NCIMB 10525) (39). This substrate has an F(MM) of 1.00 and a number-average degree of polymerization (DP_n_) of around 370, determined from its weight-average molecular weights (*M*_w_s) by size exclusion chromatography with multiangle light scattering (SEC-MALS) (*M*_n_ of 73 kDa and *M*_w_ of 146 kDa). ^13^C-1-labeled polyM was produced from the fermentation of the same strain using ^13^C-1 (99% ^13^C)-labeled d-fructose (Cambridge Isotope Laboratories, USA). This substrate was further subjected to acid hydrolysis as described previously (40, 41) and had a final DP_n_ of around 70. 5-^3^H-labeled mannuronan was produced by growing the same strain on 5-^3^H-labeled glucose (Amersham). PolyMG was produced by epimerizing polyM with AlgE4 epimerase (42) until reaction completion, obtaining a final F(G) of 0.46 and an F(GG) of 0. ^13^C-labeled polyMG was created in the same way, using ^13^C-labeled polyM. This substrate was subjected to acid hydrolysis, obtaining a DP_n_ of around 80. ^13^C-labeled oligo-G was produced by the *in vitro* epimerization of ^13^C-labeled polyM with AlgE1 epimerase and then subjecting this product to acid hydrolysis (4, 43) to obtain a final DP_n_ of around 21 and an F(G) of 0.97.

### Mutagenesis, protein production, and purification.

Plasmids encoding AlgE7 variants (Table 2) were constructed based on wild-type AlgE7 (plasmid pBG27) (12) using the QuikChange site-directed mutagenesis kit (Stratagene/Agilent) or Q5 site-directed mutagenesis (New England BioLabs), according to the instructions provided by the manufacturers. Primer sequences can be provided upon request. All cloning was performed using Escherichia coli DH5α competent cells (New England BioLabs).

**TABLE 2:** Plasmids used in this study^*a*^

Plasmid	Description	Source or reference
pTYB1	Expression vector	New England BioLabs
pBG27	pTRc99a derivative; contains the *algE7* gene	12
pKK2	pBG27 with *algE7-*E117L	This study
pRS16	pBG27 with *algE7-*E117K	This study
pRS38	pBG27 with *algE7-*D119A	This study
pRS39	pBG27 with *algE7-*D119E	This study
pRS40	pBG27 with *algE7-*D119N	This study
pKK3	pBG27 with *algE7-*Y122A	This study
pRS17	pBG27 with *algE7-*Y122F	This study
pTB95	pBG27 with *algE7-*R148G	This study
pJR4	pBG27 with *algE7-*R148K	This study
pJR3	pBG27 with *algE7-*Y149A	This study
pRS18	pBG27 with *algE7-*Y149F	This study
pRS35	pBG27 with *algE7-*Y149H	This study
pRS19	pBG27 with *algE7-*D152E	This study
pRS20	pBG27 with *algE7-*D152N	This study
pRS22	pBG27 with *algE7-*H154F	This study
pRS21	pBG27 with *algE7-*H154Y	This study
pJR1	pBG27 with *algE7-*K172L	This study
pJR2	pBG27 with *algE7-*K172R	This study
pKK5	pBG27 with *algE7-*D173A	This study
pRS23	pBG27 with *algE7-*D178E	This study
pRS24	pBG27 with *algE7-*D178N	This study
pRS25	pBG27 with *algE7-*D178R	This study
pRS26	pBG27 with *algE7-*R195L	This study
pKK7	pBG27 with *algE7-*H196A	This study
pKK10	pBG27 with *algE7-*E117K-R148G	This study
pKK11	pBG27 with *algE7*-E117K-R148G-K172L	This study
pJR5	pBG27 with *algE7-*R148K-K172L	This study
pJR6	pBG27 with *algE7-*R148K-K172R	This study
pTB113	pBG27 with *algE7-*E117K-Y122F-R148G	This study
pTB118	pBG27 with *algE7*-E117K-Y122F-K172L	This study
pTB119	pBG27 with *algE7*-E117K-Y122F-K172R	This study
pTB111	pBG27 with *algE7*-E117K-Y122F-R148G-K172L	This study
pTB112	pBG27 with *algE7*-E117K-Y122F-R148G-K172R	This study
pTB116	pBG27 with *algE7*-E117K-Y122F-Y149A	This study

a Sequences can be provided upon request.

Enzyme extracts of wild-type and mutant AlgE7 proteins used in the *A*_230_ lyase activity assay were produced in 96-well plates as previously described (27). AlgE7 variant proteins and wild-type AlgE7 were expressed and purified in two different ways. Enzymes were partially purified after expression in DH5α at 37°C by fast protein liquid chromatography on a HiTrap-Q column (GE Healthcare), using two purification buffers and a disruption buffer containing 20 mM MOPS [3-(*N*-morpholino)propanesulfonic acid] (pH 6.9) supplemented with 2 mM CaCl_2_, 2 mM CaCl_2_ plus 1 M NaCl, and 4 mM CaCl_2_, respectively. AlgE7 and R148G were purified completely by expressing the proteins in the pTYB1 vector containing a C-terminal intein tag in T7 Express competent E. coli cells (New England BioLabs) and the purifying enzyme extract by affinity chromatography using the Impact-CN system (New England BioLabs). Enzymes were eluted in HEPES buffer (20 mM HEPES, 5 mM CaCl_2_, 500 mM NaCl [pH 6.9]) and dialyzed in a solution containing 5 mM HEPES and 5 mM CaCl_2_ to remove dithiothreitol (DTT) and NaCl from the purification buffer, before they were freeze-dried for storage.

Protein concentrations were estimated by the Bio-Rad protein assay (44) or from the absorbance at 280 nm using a NanoDrop spectrophotometer and extinction coefficients calculated with the ExPASy ProtParam online tool (45).

### Activity assays.

Two assays were performed for the AlgE7 variants, the tritium assay and the *A*_230_ assay. The tritium assay was performed on partially purified enzymes by measuring the liberation of ^3^H from [5-^3^H]mannuronan, essentially as described previously (32). The alginate substrate and product were precipitated and removed from the sample before measuring the liberated ^3^H. Incubation with excess enzyme or prolonged incubation with a lyase will prevent efficient precipitation of the substrate due to the resulting short alginate oligomers, leading to an overestimation of the activity. Thus, appropriate enzyme amounts and incubation times were identified to optimize this assay. The reaction buffer contained 20 mM MOPS (pH 6.9) and 2 mM CaCl_2_, and reaction mixtures were incubated at 37°C. The *A*_230_ assay was performed for crude enzyme extracts of variants by adding 20 μL of extracts to total reaction mixtures of 250 μL with 1 mg/mL polyM in 50 mM Tris buffer with 2.5 mM CaCl_2_. The absorbance was measured in Costar UV microplates every 5 min for 18 h at 25°C in a Spectramax ABS Plus microplate reader.

For AlgE7 purified using the Impact-CN protocol, the *A*_230_ assay was performed in 50 mM Tris buffer with various concentrations of NaCl (0, 50, 150, and 300 mM) and CaCl_2_ (0, 1, and 3 mM), various pHs (6, 7, and 8), and various concentrations of the polyM substrate (0.125 and 1 mg/mL), with a constant enzyme concentration of 0.3 μM. The reactions were performed in Costar UV 96-well plates, measuring the absorbance at 230 nm every third minute, for 7 to 18 h (until an endpoint was reached) at 25°C. All reaction mixes contained 0.16 mM CaCl_2_ originating from the enzyme buffer. Each combination was performed twice.

### Product patterns with NMR and HPAEC-PAD.

A total of 0.25% (wt/vol) (2.5 mg/mL) polyM or polyMG was dissolved in 5 mM HEPES buffer with 2.5 mM CaCl_2_ (pH 6.9). AlgE7 and R148G purified by the Impact-CN protocol were added at a 1:300 (wt/wt) ratio to the substrate, to final concentrations of about 8.3 μg/mL. The reactions were left at 25°C for different time intervals. Reactions were stopped by the addition of EDTA to a final concentration of 5 mM, and the mixtures were heated at 90°C for 15 min and subsequently freeze-dried. The samples were then dissolved in 600 μL D_2_O with 20 μL 0.3 M TTHA (triethylenetetraamine-hexaacetate) (pH 7) as a calcium chelator and 2.5 μL 1% TSP [3-(trimethylsilyl)-propionic-2,2,3,3-d_4_ acid sodium salt] as a reference. NMR acquisition was done on a Bruker Avance III HD 400-MHz instrument equipped with a 5-mm SmartProbe (Bruker BioSpin AG, Fällenden, Switzerland) at the NV-NMR Centre/Norwegian NMR Platform (NNP) at the Norwegian University of Science and Technology (NTNU), with a 30° flip angle pulse, 64 scans, and a spectral width of 10 ppm. All spectra were recorded at 90°C to decrease sample viscosity for increased resolution and to move the solvent peak away from the chemical shift region of alginate. Integrations of spectra were done in TopSpin, and peaks were assigned as described previously (12, 46, 47). Sequential parameters were calculated according to the procedures in the supplemental material.

HPAEC-PAD was performed for the last time points of the AlgE7 reaction on polyM and polyMG (60 h), using oligomeric standards created by the degradation of polyM and polyMG by an M-lyase from Haliotis tuberculata and by the degradation of polyG by the G-lyase AlyA from Klebsiella pneumoniae as previously described (43, 48).

### Time-resolved NMR.

Time-resolved ^13^C NMR experiments following the epimerization reaction were performed as previously described (14, 28), using 10 mg/mL of ^13^C_1_-enriched polyM, oligo-G, and polyMG. AlgE7 and R148G purified by the Impact-CN protocol were added to final concentrations of 2.8 to 2.9 μM. The buffer contained 10 mM MOPS (pH 6.9), 75 mM NaCl, and 2.5 mM CaCl_2_ in 99.8% D_2_O. For the wild type, the same experiment was also performed with various concentrations of NaCl (0, 50, 75, 150, and 300 mM) and calcium (0, 1, 2.5, and 3 mM). After the completion of the time-resolved experiment, a ^13^C-^1^H HSQC spectrum was recorded for the assignment of the products formed during the enzymatic reaction. The time-resolved data were acquired at 25°C using a Bruker Avance III HD 800-MHz spectrometer (Bruker BioSpin AG, Fällenden, Switzerland) equipped with a 5-mm Z-gradient CP-TCI (H/C/N) cryogenic probe at the NV-NMR Centre/NNP at NTNU. All NMR spectra were recorded using TopSpin version 3.5.7 and processed using TopSpin version 4.0.8 software (Bruker BioSpin).

### Data availability.

All data are contained within the article and the supplemental material.
