# Supplementary material for: Mechanistic Basis for Understanding the Dual Activities of the Bifunctional Azotobacter vinelandii Mannuronan C-5-Epimerase and Alginate Lyase AlgE7
Source: Appl Environ Microbiol. 2022 Feb 8;88(3):e01836-21. doi: 10.1128/aem.01836-21 (PMC8824271; doi:10.1128/aem.01836-21)
Supplement: Supplemental file 1 — Fig. S1 to S8, Tables S1 to S3. Download aem.01836-21-s0001.pdf, PDF file, 1.8 MB [file aem.01836-21-s0001.pdf]

Supporting Information

A mechanistic basis for understanding the dual activities of the bifunctional *Azotobacter vinelandii* mannuronan C-5 epimerase and alginate lyase AlgE7

Margrethe Gaardløs[1], Tonje Marita Bjerkan Heggeset[1,2], Anne Tøndervik[2], David Teze[3,4], Birte Svensson[3], Helga Ertesvåg[1], Håvard Sletta[2] and Finn Lillelund Aachmann[1]*.

[1]Norwegian Biopolymer Laboratory (NOBIPOL), Department of Biotechnology and Food Science, NTNU Norwegian University of Science and Technology, Sem Sælands vei 6/8, N-7491 Trondheim, Norway

[2]Department of Biotechnology and Nanomedicine, SINTEF Industry, Postboks 4760 Torgarden, N-7465 Trondheim, Norway.

[3]Department of Biotechnology and Biomedicine, Technical University of Denmark, DK-2800 Kgs. Lyngby, Denmark.

[4]Novo Nordisk Foundation Center for Biosustainability, Technical University of Denmark, DK-2800 Kgs. Lyngby, Denmark

Figure S1: HSQC-spectra
Figure S2: Time-resolved ^{13}C NMR, AlgE7 and oligoG
Figure S3: ^{1}H NMR, calculation of sequential parameters
Figure S4: ^{1}H NMR-spectra
Figure S5: HPAEC-PAD chromatograms
Figure S6: Protein sequences
Figure S7: Curves from factorial A230 lyase activity assay
Figure S8: End point values from AlgE7 factorial A230 lyase activity assay

Table S1: Fraction of signals from ^{1}H NMR of AlgE7 and R148G at different time points
Table S2: Fraction of signals from ^{1}H NMR of variants
Table S3: Slopes and end point values from AlgE7 factorial A230 lyase activity assay

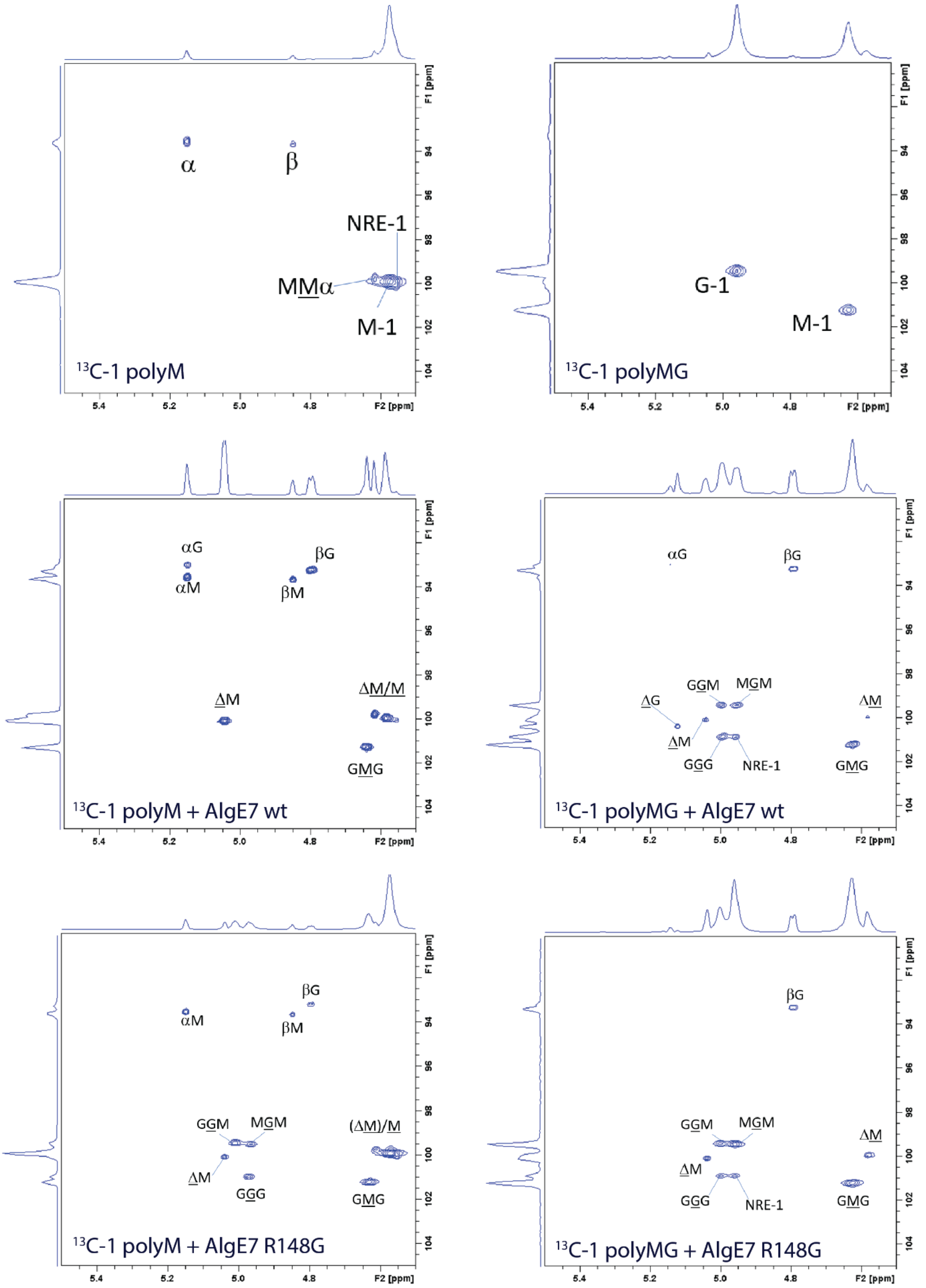

Figure S1: HSQC spectra of reaction products from 2.8–2.9 µM AlgE7 wild type and R148G on 10 mg/ml polyM or polyMG, in buffer containing 75 mM NaCl and 2.5 mM NaCl, pH 7.0. The spectra were recorded at the end of time-resolved ^{13}C-NMR experiments, and assigned as shown, according to previous work (Grasdalen et al. 1981; Grasdalen 1983; Holtan, Zhang, et

al. 2006). Triads or dyads are indicated in the spectra, and the residue giving rise to the signal is underlined. NRE-1 denotes the signal from carbon 1 at the non-reducing end.

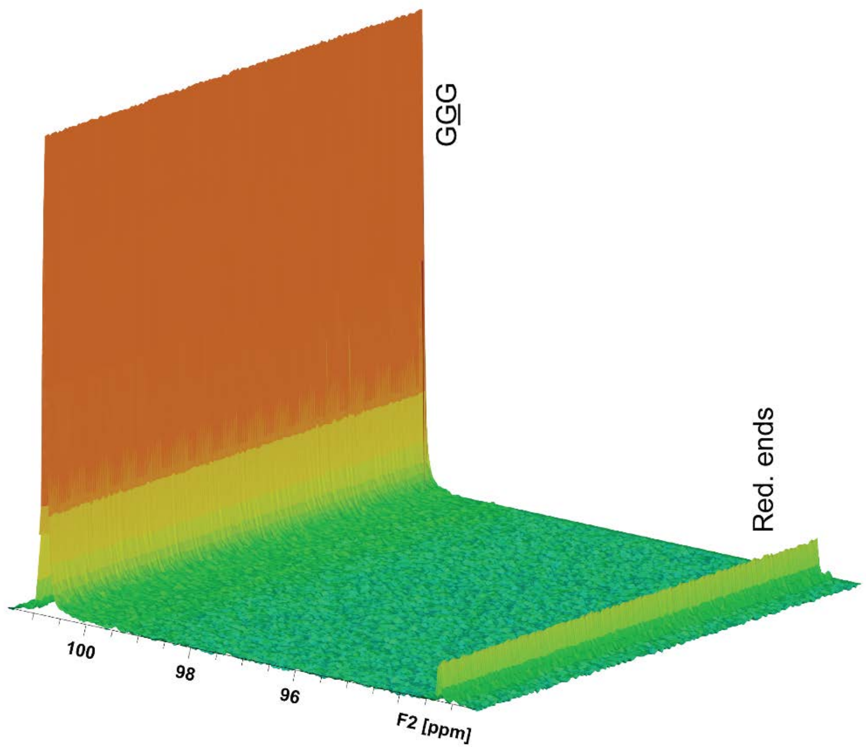

Figure S2: OligoG substrate incubated with AlgE7 produced no change in ^{13}C time resolved NMR spectra after 16 hours. The same test was performed with R148G, and as the results were identical only the spectrum for the wild type is shown.

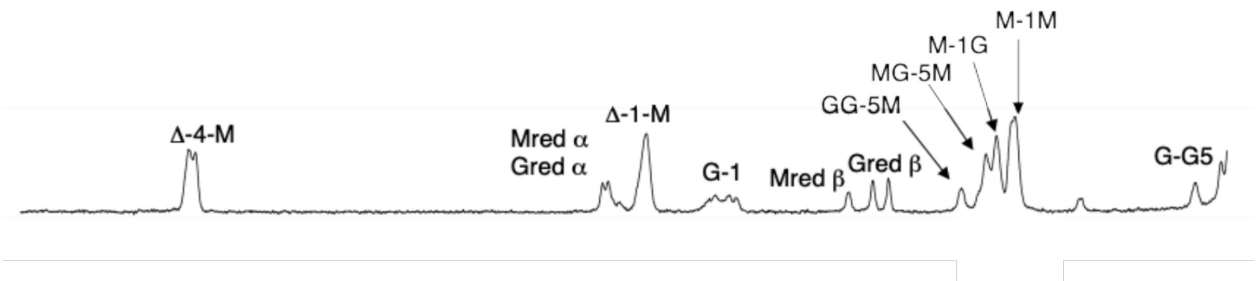

Figure S3: Example chromatogram showing the anomeric region of polyM epimerized by the AlgE7 variant Y122F. The G-1 peak comes from all internal G-residues, but for some of the active lyases it almost disappeared. However, there were clearly internal G-residues present, seen from the peaks of GGM-5, MGM-5 and MG-1. Due to poor baseline separation, the peaks for MGM-5 and MG-1 were difficult to integrate separately. Nevertheless, this was done, as the two peaks contain some of the information required to calculate the amount of total G- and M-residues in the sample. The results are not as robust as they would be for the maximum averaging procedure that is possible to do for epimerized alginate with high DP_n, but they can still be used for qualitative comparisons of different time points and variants. Calculations of sequential parameters of epimerized and lyased alginate were done using the following relationships between signal intensities:

1. I(total) = I(GGM-5) + I(MGM-5) + I(MG-1) + I(MM-1) + I(GG-5) + I(Δ) + I(M_{red}) + I(G_{red})
2. F(G_{int}) = I(G-1)/I(total)
3. F(GGM) = I(GGM-5) / I(total)
4. F(MM) = I(MM-1) / I(total)
5. F(GG) = I(GG-5) / I(total)
6. F(Δ) = I(ΔM-1) / I(total)
7. F(G_{red}) = (I(G_{red}β) + I((G_{red}β) · 0.2)) / I(total)
8. F(M_{red}) = (M_{red}β + (M_{red}β· 2.2)) / I(total)

9. F(G) = (I(GGM-5) + I(MGM-5) + I(GG-5) + I(G_{red})) / I(total)
10. F(M) = (I(MG-1) + I(MM-1) + I(M_{red})) / I(total)
11. F(M_{int}) = F(M)-F(M_{red})

As the signals for M and G reducing ends in α-configuration are not resolved, the known relationships of G_{red}α being 0.2 of G_{red}β, and M_{red}α being 2.2 times more than M_{red}β, are used (Heyraud et al. 1996).

Fractions were calculated by dividing the relevant intensity by the total intensity of all peaks. Number average degree of polymerization (DP_n) was calculated as following:

$$DP_n = \frac{I(total)}{I(M_{red}) + I(G_{red})}$$

Based on these calculations, we obtained the values listed in Table S2 from incubating partially purified protein extracts of AlgE7 wild type and variants with 1 mg/ml polyM for 24 hours. In

Table S1 the data for purified wild type AlgE7 and R148G incubated with 2.5 mg/ml substrate (enzyme:substrate ratio 1:300, w/w) at different reaction time points is listed.

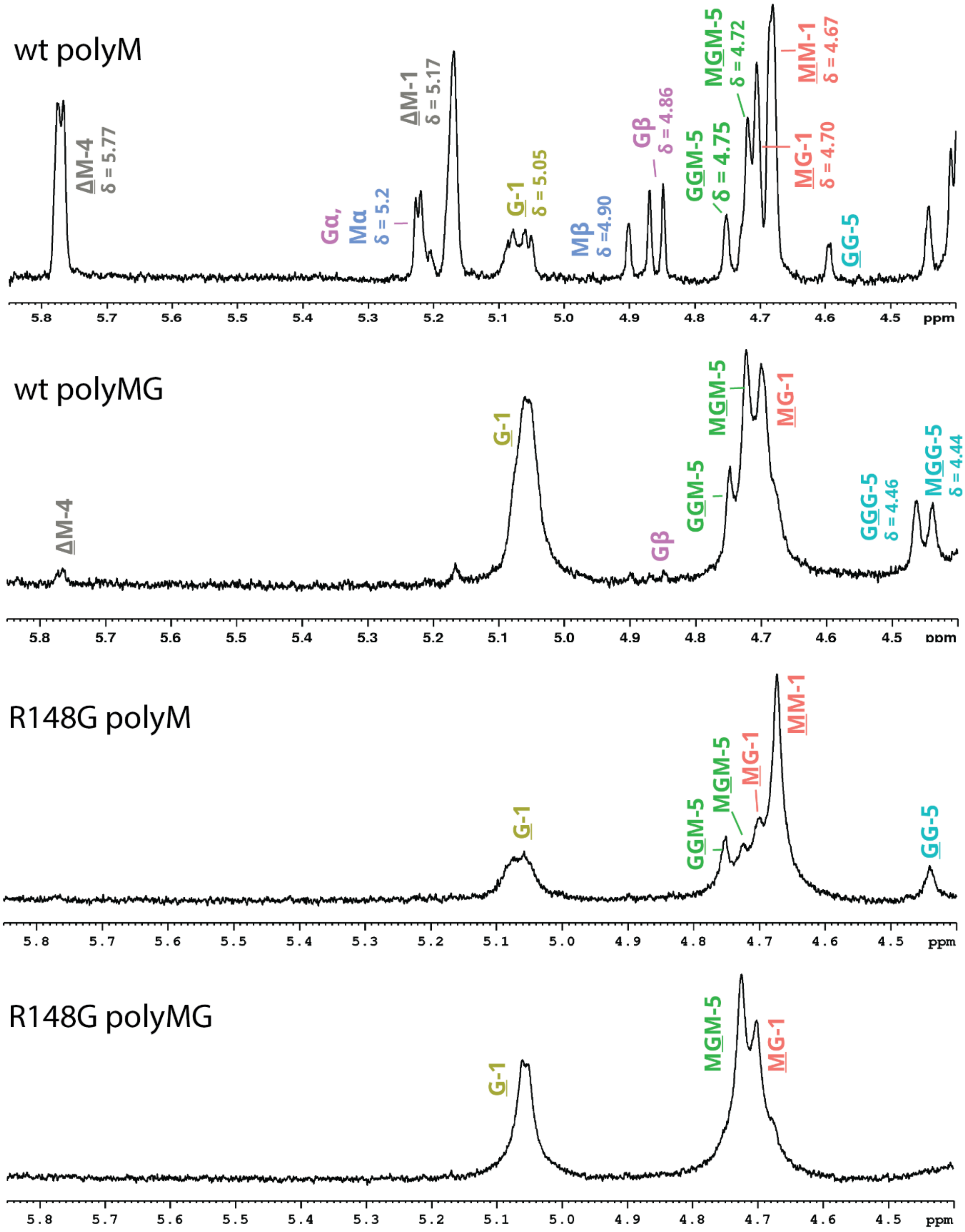

Figure S4: ^{1}H-NMR of reactions of AlgE7 (wt) and R148G on polyM and polyMG, with no added NaCl, after 60 h of enzyme reaction. Compare with Figures 2 and 5 for the results with 75 mM NaCl added.

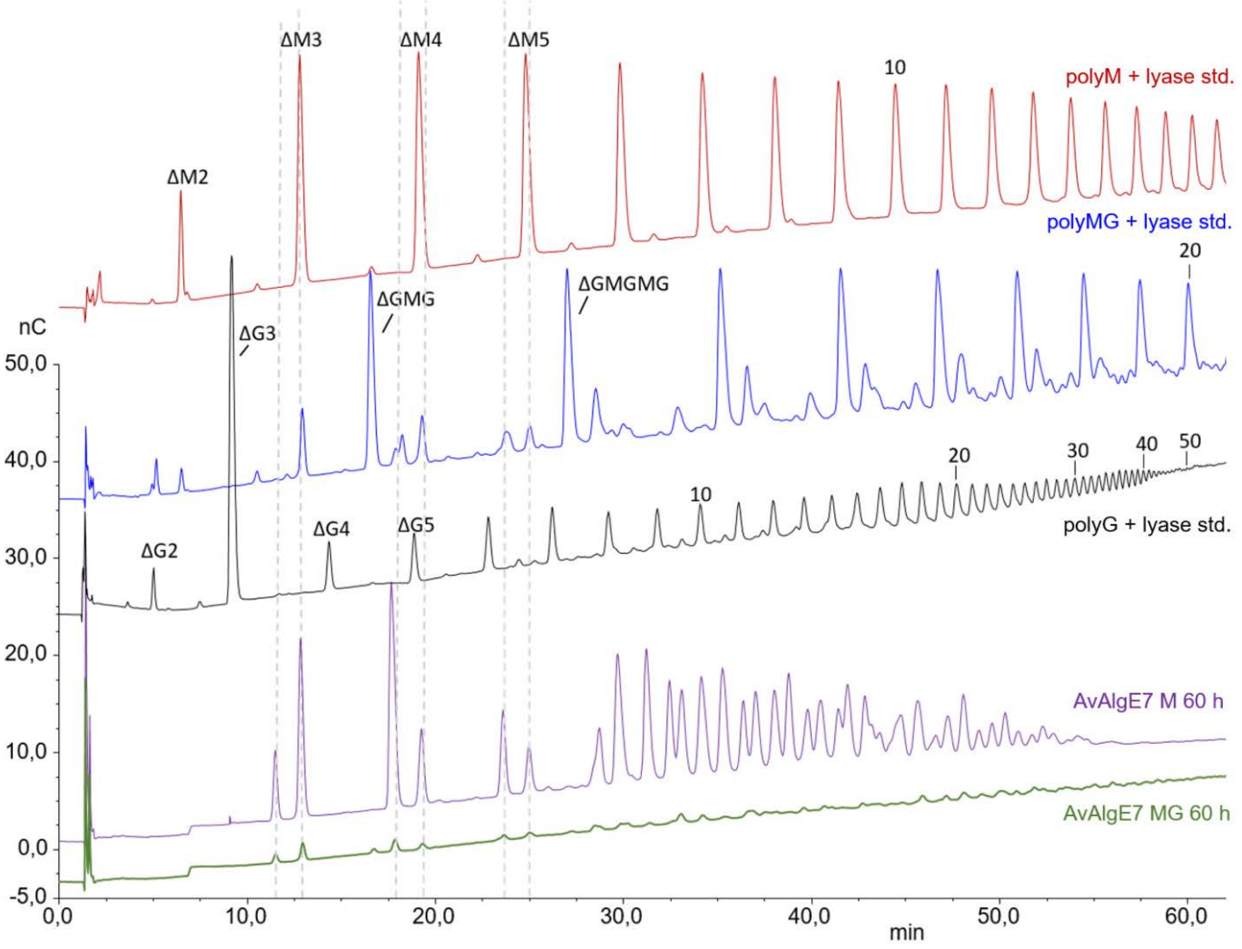

Figure S5: HPAEC-PAD chromatogram of the products produced by AlgE7 after incubation of 60 h on polyM (purple) and polyMG (green). Standards of M-lyase from *Haliotis tubercalata* on polyM and polyMG, and G-lyase AlyA from *Klebsiella pneumoniae* on polyG, are shown in red, blue and black lines, respectively. Relevant, annotated peaks of the standards are marked (Aarstad et al. 2012).

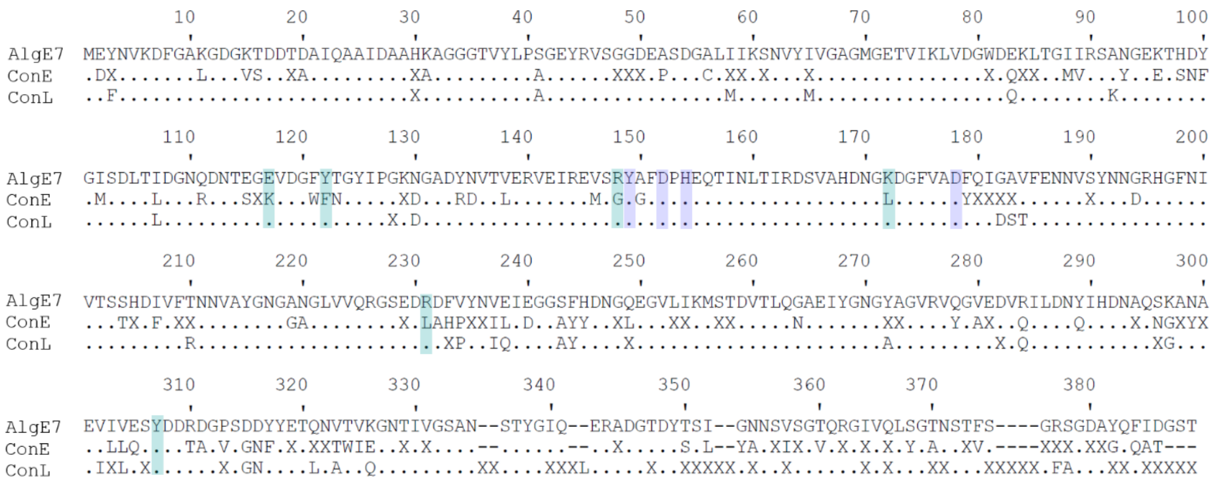

Figure S6: Sequence alignment of AlgE7 and consensus sequences of the nine A-modules that are pure epimerases (ConE) and the three A-modules that are bifunctional lyases/epimerases (conL) from *A. vinelandii* and *A. chroococcum*. The active site residues are highlighted in purple, and residues mutated in this study are highlighted in blue. An excerpt of this is also shown in Figure 3A.

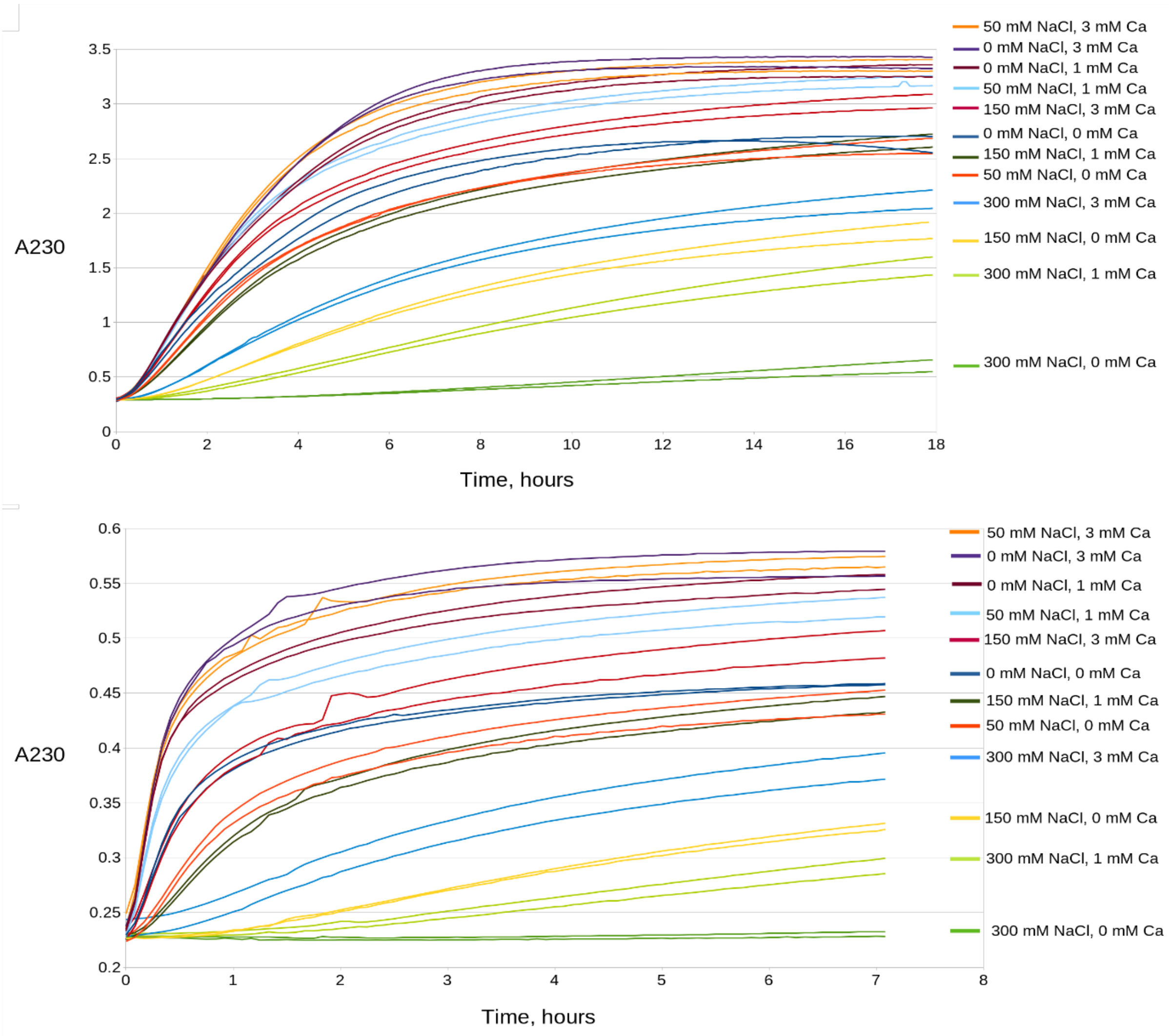

Figure S7: Lyase activity over time measured from the spectrophotometric A230 assay for AlgE7 on two different concentrations of polyM and different concentrations of NaCl and $CaCl_2$. Similar curves were obtained at pH 6 and 8, and for the amino acid replacement variants. Initial, linear regions of the curves were used to calculate the slopes, listed in Table 1 as percentages of wild type for the enzyme variants, and in Table S3 for the factorial assay together with values at end point (with subtracted start values). This figure is meant to illustrate the general shape of the curves at the two different substrate concentrations (1 mg/ml in the top plot and 0.125 mg/ml in the bottom plot), and all differences are illustrated more clearly from the slopes plotted in Figures 6 and S8, and the values at end point shown in Figure S8.

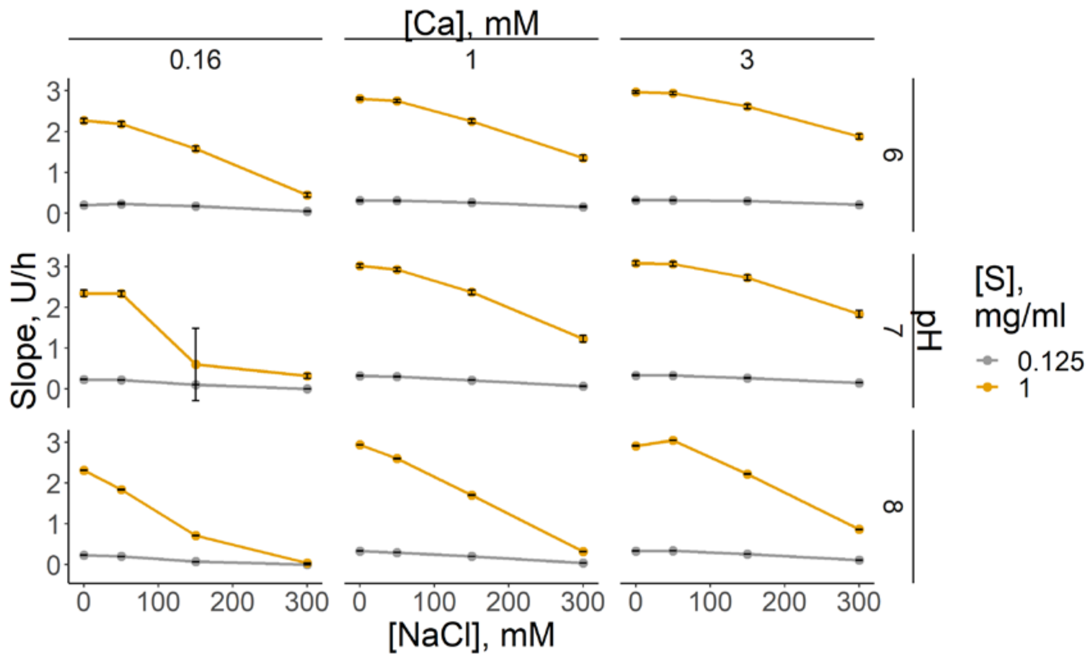

Figure S8: Mean end point values of factorial lyase assay of the wild type AlgE7. Points are averages of two repetitions, and error bars show the range of values for the repetitions.

Table S1: Fraction of signals from end point ^{1}H NMR of wild type AlgE7 and R148G at different reaction time points, analysed with ^{1}H NMR.

Sample	Time (h)	F(G)	F(M)	F(GG)	F(MM)	F(G$_{int}$)	F(M$_{int}$)	F(GGM)	F(Δ)	F(G$_{red}$)	F(M$_{red}$)	DPn
Wt + polyM	0	0.00	1.00	0.00	1.00	0.00	1.00	0.00	0.00	0.00	0.00	NaN
0 mM NaCl	6	0.18	0.80	0.04	0.67	0.12	0.80	0.04	0.02	0.01	0.01	78.09
	12	0.22	0.74	0.05	0.53	0.18	0.70	0.06	0.04	0.02	0.04	17.18
	24	0.25	0.65	0.07	0.38	0.19	0.58	0.07	0.10	0.05	0.06	9.19
	48	0.30	0.55	0.09	0.31	0.16	0.49	0.06	0.15	0.06	0.07	7.89
	60	0.30	0.50	0.06	0.26	0.11	0.41	0.04	0.20	0.09	0.09	5.56
Wt + polyMG	0	0.49	0.51	0.03	0.12	0.49	0.51	0.00	0.00	0.00	0.00	NaN
0 mM NaCl	6	0.57	0.42	0.03	0.00	0.53	0.40	0.13	0.01	0.01	0.01	NaN
	12	0.53	0.47	0.05	0.00	0.56	0.45	0.11	0.01	0.01	0.02	NaN
	24	0.58	0.40	0.09	0.00	0.59	0.39	0.14	0.02	0.01	0.01	54.81
	48	0.58	0.40	0.13	0.00	0.60	0.39	0.14	0.02	0.01	0.01	44.85
	60	0.61	0.37	0.16	0.00	0.65	0.35	0.14	0.02	0.01	0.02	29.98
R148G + polyM	0	0.00	1.00	0.00	1.00	0.00	1.00	0.00	0.00	0.00	0.00	NaN
0 mM NaCl	6	0.17	0.83	0.01	0.71	0.07	0.80	0.06	0.01	0.02	0.02	NaN
	12	0.18	0.82	0.02	0.69	0.10	0.82	0.07	0.00	0.01	0.01	59.50
	24	0.22	0.77	0.04	0.62	0.16	0.76	0.08	0.01	0.01	0.02	40.40
	48	0.27	0.71	0.05	0.54	0.21	0.70	0.12	0.01	0.01	0.01	43.25
	60	0.27	0.72	0.06	0.53	0.24	0.70	0.11	0.01	0.01	0.02	33.06
R148G + polyMG	0	0.49	0.51	0.00	0.12	0.49	0.51	0.00	0.00	0.00	0.00	NaN
0 mM NaCl	6	0.56	0.44	0.04	0.12	0.48	0.44	0.10	0.00	0.00	0.00	NaN
	12	0.53	0.47	0.03	0.14	0.48	0.47	0.10	0.00	0.00	0.00	NaN
	24	0.54	0.46	0.03	0.13	0.48	0.46	0.10	0.00	0.00	0.00	NaN
	48	0.54	0.46	0.03	0.13	0.48	0.46	0.10	0.00	0.00	0.00	NaN
	60	0.55	0.45	0.03	0.12	0.49	0.45	0.10	0.00	0.00	0.00	NaN
Wt + polyM	0	0.00	1.00	0.00	1.00	0.00	1.00	0.00	0.00	0.00	0.00	NaN
75 mM NaCl	6	0.15	0.84	0.03	0.72	0.09	0.84	0.04	0.01	0.00	0.00	352.53
	12	0.20	0.78	0.06	0.63	0.14	0.77	0.05	0.02	0.01	0.01	71.61
	24	0.23	0.71	0.07	0.50	0.19	0.68	0.07	0.06	0.02	0.02	21.74
	48	0.28	0.60	0.09	0.35	0.16	0.55	0.07	0.12	0.05	0.05	10.07
	60	0.28	0.58	0.07	0.32	0.17	0.51	0.06	0.14	0.07	0.06	7.62
Wt + polyMG	0	0.54	0.46	0.03	0.12	0.49	0.46	0.00	0.00	0.00	0.00	NaN
75 mM NaCl	6	0.61	0.41	0.10	0.08	0.55	0.41	0.10	0.02	0.01	0.00	92.02
	12	0.54	0.44	0.12	0.08	0.58	0.43	0.14	0.03	0.02	0.01	38.16
	24	0.56	0.41	0.16	0.06	0.61	0.40	0.15	0.03	0.02	0.00	51.86
	48	0.57	0.40	0.21	0.06	0.63	0.39	0.15	0.04	0.02	0.01	32.37
	60	0.59	0.37	0.22	0.05	0.64	0.37	0.16	0.04	0.02	0.01	41.26
R148G + polyM	0	0.00	1.00	0.00	1.00	0.00	1.00	0.00	0.00	0.00	0.00	NaN
75 mM NaCl	6	0.11	0.89	-0.02	0.83	0.03	0.89	0.05	0.00	0.00	0.00	NaN
	12	0.14	0.85	-0.02	0.73	0.07	0.85	0.07	0.00	0.00	0.00	NaN
	24	0.18	0.82	0.00	0.70	0.09	0.82	0.08	0.00	0.00	0.00	NaN
	48	0.22	0.76	0.04	0.61	0.18	0.75	0.10	0.01	0.01	0.02	33.34
	60	0.25	0.75	0.05	0.61	0.17	0.75	0.10	0.00	0.00	0.00	NaN

R148G + polyMG	0	0.49	0.51	0.00	0.12	0.49	0.51	0.00	0.00	0.00	0.00	NaN
75 mM NaCl	6	0.56	0.45	0.03	0.09	0.50	0.45	0.07	0.00	0.00	0.00	NaN
	12	0.53	0.48	0.04	0.14	0.48	0.48	0.08	0.00	0.00	0.00	NaN
	24	0.58	0.42	0.05	0.10	0.51	0.42	0.09	0.02	0.00	0.00	NaN
	48	0.59	0.42	0.06	0.10	0.52	0.42	0.10	0.01	0.01	0.00	82.38
	60	0.59	0.42	0.07	0.10	0.52	0.42	0.10	0.02	0.01	0.00	80.84

Table S2: Fraction of signals from end point ^{1}H NMR of all variants tested in this study. G_{red} and M_{red} are residues at reducing ends, and G_{int} are internal G-residues.

Enzyme	F(G)	F(M)	F(GG)	F(MM)	F(G_{int})	F(M_{int})	F(GGM)	F(Δ)	F(G_{red})	F(M_{red})	DPn
Wt	0.24	0.47	0.01	0.22	0.13	0.34	0.00	0.29	0.11	0.14	4.02
Wt	0.25	0.47	0.01	0.21	0.14	0.32	0.01	0.28	0.11	0.15	3.86
E117L	0.30	0.45	0.06	0.23	0.20	0.35	0.02	0.25	0.11	0.09	4.94
E117L	0.30	0.49	0.05	0.25	0.20	0.40	0.04	0.21	0.10	0.09	5.39
E117K	0.31	0.52	0.07	0.26	0.21	0.43	0.07	0.17	0.10	0.09	5.17
E117K	0.30	0.49	0.05	0.26	0.19	0.40	0.04	0.20	0.11	0.09	4.96
D119A	0.00	1.00	0.00	1.00	0.00	1.00	0.00	0.00	0.00	0.00	NaN
D119A	0.00	1.00	0.00	1.00	0.00	1.00	0.00	0.00	0.00	0.00	NaN
D119E	0.26	0.62	0.08	0.39	0.21	0.56	0.07	0.11	0.05	0.06	9.18
D119E	0.26	0.67	0.07	0.48	0.22	0.64	0.07	0.07	0.04	0.03	14.10
D119N	0.00	1.00	0.00	1.00	0.00	1.00	0.00	0.00	0.00	0.00	NaN
D119N	0.00	1.00	0.00	1.00	0.00	1.00	0.00	0.00	0.00	0.00	NaN
Y122A	0.00	1.00	0.00	1.00	0.00	1.00	0.00	0.00	0.00	0.00	NaN
Y122A	0.00	1.00	0.00	1.00	0.00	1.00	0.00	0.00	0.00	0.00	NaN
Y122F	0.32	0.51	0.08	0.26	0.22	0.45	0.07	0.17	0.10	0.05	6.54
Y122F	0.32	0.50	0.06	0.24	0.21	0.42	0.06	0.18	0.11	0.08	5.25
R148G	0.20	0.80	0.02	0.69	0.18	0.79	0.07	0.00	0.01	0.01	51.60
R148G	0.31	0.67	0.07	0.51	0.30	0.66	0.12	0.02	0.01	0.01	45.41
R148K	0.34	0.62	0.13	0.42	0.32	0.60	0.11	0.04	0.02	0.02	25.61
R148K	0.30	0.66	0.08	0.46	0.27	0.64	0.10	0.04	0.03	0.02	21.62
K172L	0.29	0.49	0.03	0.25	0.18	0.39	0.03	0.22	0.11	0.09	4.95
K172L	0.37	0.47	0.12	0.24	0.26	0.39	0.05	0.17	0.11	0.08	5.29
K172R	0.25	0.46	0.00	0.21	0.13	0.32	0.00	0.29	0.12	0.14	3.90
K172R	0.24	0.47	0.01	0.22	0.12	0.33	0.00	0.29	0.12	0.13	3.93
D173A	0.00	1.00	0.00	1.00	0.00	1.00	0.00	0.00	0.00	0.00	NaN
D173A	0.00	1.00	0.00	1.00	0.00	1.00	0.00	0.00	0.00	0.00	NaN
E117K + R148G	0.36	0.61	0.13	0.40	0.35	0.60	0.13	0.02	0.01	0.02	35.38
E117K + R148G	0.34	0.65	0.08	0.47	0.32	0.64	0.13	0.01	0.02	0.01	29.45
E117K + R148G + K127L	0.39	0.58	0.12	0.37	0.37	0.56	0.16	0.03	0.03	0.02	20.23
E117K + R148G + K127L	0.38	0.60	0.12	0.39	0.36	0.58	0.15	0.02	0.02	0.02	26.57
R148G + K172L	0.25	0.75	0.08	0.62	0.25	0.75	0.09	0.00	0.00	0.00	NaN
R148G + K172L	0.19	0.81	0.04	0.68	0.19	0.81	0.08	0.00	0.00	0.00	NaN
R148G + K172R	0.32	0.66	0.06	0.48	0.31	0.65	0.13	0.02	0.01	0.01	49.25
R148G + K172R	0.31	0.68	0.08	0.51	0.30	0.68	0.12	0.01	0.01	0.00	140.97
E117K + Y122F + R148G	0.40	0.59	0.15	0.38	0.40	0.59	0.17	0.01	0.00	0.00	NaN
E117K + Y122F + R148G	0.39	0.60	0.11	0.41	0.38	0.60	0.17	0.01	0.01	0.00	85.36
E117K + Y122F + K172L	0.39	0.50	0.16	0.25	0.34	0.49	0.14	0.11	0.05	0.00	18.96
E117K + Y122F + K172L	0.39	0.50	0.16	0.25	0.33	0.49	0.13	0.11	0.06	0.01	13.85
E117K + Y122F + K172R	0.39	0.54	0.15	0.29	0.35	0.50	0.13	0.07	0.04	0.04	12.56
E117K + Y122F + K172R	0.38	0.57	0.14	0.34	0.34	0.54	0.14	0.06	0.04	0.02	17.20
E117K + Y122F + R148G + K172L	0.45	0.54	0.21	0.35	0.45	0.54	0.15	0.01	0.00	0.00	NaN
E117K + Y122F + R148G + K172L	0.35	0.63	0.11	0.45	0.35	0.63	0.15	0.02	0.00	0.00	NaN

E117K + Y122F + R148G + K172R	0.33	0.65	0.11	0.50	0.31	0.64	0.12	0.02	0.01	0.01	46.73
E117K + Y122F + R148G + K172R	0.28	0.71	0.10	0.56	0.28	0.71	0.10	0.01	0.00	0.00	NaN

Table S3: Calculated slopes and end point values (initial value subtracted) for all the lyase activity experiments performed for wild type AlgE7 at three different pH values, two different substrate concentrations, four different salt concentrations, and three different calcium concentrations. Each experiment was repeated twice, and values from both experiments are shown.

	mM		1 mg/ml [S]				0.125 mg/ml]S]			
pH	[NaCl]	[Ca]	Slope		End value		Slope		End value	
6	0	0	0.25	0.24	2.49	2.61	0.22	0.20	0.42	0.44
	50	0	0.24	0.25	2.41	2.54	0.16	0.17	0.45	0.48
	150	0	0.09	0.09	1.80	1.92	0.00	0.01	0.39	0.42
	300	0	0.00	0.01	0.67	0.78	0.00	0.00	0.27	0.29
	0	1	0.32	0.32	3.06	3.11	0.30	0.32	0.53	0.56
	50	1	0.34	0.34	3.00	3.07	0.33	0.32	0.53	0.56
	150	1	0.25	0.25	2.49	2.59	0.11	0.11	0.49	0.50
	300	1	0.04	0.05	1.57	1.71	0.01	0.01	0.37	0.41
	0	3	0.35	0.34	3.24	3.30	0.32	0.33	0.54	0.57
	50	3	0.39	0.39	3.21	3.29	0.37	0.38	0.54	0.57
	150	3	0.35	0.36	2.85	2.95	0.23	0.24	0.53	0.54
	300	3	0.14	0.15	2.10	2.23	0.02	0.01	0.43	0.45
7	0	0	0.55	0.48	2.55	2.70	0.24	0.23	0.46	0.46
	50	0	0.41	0.40	2.55	2.68	0.09	0.09	0.43	0.45
	150	0	0.07	0.07	1.77	0.00	0.00	0.00	0.33	0.33
	300	0	0.00	0.01	0.55	0.66	0.00	0.00	0.23	0.23
	0	1	0.65	0.67	3.25	3.36	0.49	0.50	0.54	0.56
	50	1	0.61	0.63	3.17	3.26	0.40	0.41	0.52	0.54
	150	1	0.33	0.34	2.61	2.72	0.06	0.06	0.43	0.45
	300	1	0.03	0.04	1.43	1.60	0.00	0.00	0.29	0.30
	0	3	0.63	0.64	3.32	3.43	0.51	0.52	0.56	0.58
	50	3	0.62	0.63	3.30	3.41	0.49	0.51	0.57	0.57
	150	3	0.50	0.53	2.96	3.09	0.22	0.22	0.48	0.51
	300	3	0.13	0.14	2.04	2.21	0.01	0.01	0.37	0.40
8	0	0	0.39	0.38	2.61	2.60	0.14	0.15	0.47	0.48
	50	0	0.17	0.16	2.14	2.12	0.00	0.01	0.44	0.44
	150	0	0.01	0.01	1.00	0.99	0.01	0.00	0.32	0.32
	300	0	0.00	0.00	0.33	0.33	0.00	0.00	0.24	0.24
	0	1	0.62	0.63	3.22	3.22	0.43	0.42	0.57	0.57
	50	1	0.49	0.49	2.88	2.90	0.22	0.21	0.54	0.54
	150	1	0.10	0.10	2.00	1.98	0.02	0.02	0.45	0.44
	300	1	0.01	0.01	0.62	0.61	0.01	0.01	0.28	0.28
	0	3	0.54	0.53	3.23	3.21	0.36	0.36	0.58	0.58
	50	3	0.58	0.57	3.37	3.35	0.48	0.48	0.58	0.58
	150	3	0.29	0.28	2.53	2.51	0.05	0.05	0.49	0.50
	300	3	0.02	0.02	1.16	1.15	0.01	0.01	0.35	0.35

References

Aarstad, O. A., Tøndervik, A., Sletta, H., and Skjåk-Bræk, G. (2012) Alginate sequencing: An analysis of block distribution in alginates using specific alginate degrading enzymes. *Biomacromolecules*. **13**, 106–116.

Grasdalen, H., Larsen, B., and Smidsrød, O. (1981) 13C-n.m.r. studies of monomeric composition and sequence in alginate. *Carbohydrate Research*. **89**, 179–191.

Grasdalen, H. (1983) High-field, 1H-n.m.r. spectroscopy of alginate: sequential structure and linkage conformations. *Carbohydrate Research*. **118**, 255–260.

Heyraud, A., Gey, C., Leonard, C., Rochas, C., Girond, S., and Kloareg, B. (1996) NMR spectroscopy analysis of oligoguluronates and oligomannuronates prepared by acid or enzymatic hydrolysis of homopolymeric blocks of alginic acid. Application to the determination of the substrate specificity of Haliotis tuberculata alginate lyase. *Carbohydrate Research*. **289**, 11–23.

Holtan, S., Zhang, Q., Strand, W., and Skjåk-Bræk, G. (2006) Characterization of the hydrolysis mechanism of polyalternating alginate in weak acid and assignment of the resulting MG-oligosaccharides by NMR spectroscopy and ESI-mass spectrometry. *Biomacromolecules*. **7**, 2108-2121.
